# Stochastic multi-scale models of competition within heterogeneous cellular populations: Simulation methods and mean-field analysis

**DOI:** 10.1016/j.jtbi.2016.07.028

**Published:** 2016-10-21

**Authors:** Roberto de la Cruz, Pilar Guerrero, Fabian Spill, Tomás Alarcón

**Affiliations:** a Centre de Recerca Matemàtica, Edifici C, Campus de Bellaterra, 08193 Bellaterra, Barcelona, Spain; b Departament de Matemàtiques, Universitat Autònoma de Barcelona, 08193 Bellaterra, Barcelona, Spain; c Department of Mathematics, University College London, Gower Street, London WC1E 6BT, UK; d Department of Mechanical Engineering, Massachusetts Institute of Technology, 77 Massachusetts Avenue, Cambridge, MA 02139, USA; e Department of Biomedical Engineering, Boston University, 44 Cummington Street, Boston, MA 02215, USA; f ICREA, Pg. Lluís Companys 23, 08010 Barcelona, Spain; g Barcelona Graduate School of Mathematics (BGSMath), Barcelona, Spain

**Keywords:** Multi-scale modelling, Stochastic population dynamics, Cell cycle, Radiotherapy, Scaling laws

## Abstract

We propose a modelling framework to analyse the stochastic behaviour of heterogeneous, multi-scale cellular populations. We illustrate our methodology with a particular example in which we study a population with an oxygen-regulated proliferation rate. Our formulation is based on an age-dependent stochastic process. Cells within the population are characterised by their age (i.e. time elapsed since they were born). The age-dependent (oxygen-regulated) birth rate is given by a stochastic model of oxygen-dependent cell cycle progression. Once the birth rate is determined, we formulate an age-dependent birth-and-death process, which dictates the time evolution of the cell population. The population is under a feedback loop which controls its steady state size (carrying capacity): cells consume oxygen which in turn fuels cell proliferation. We show that our stochastic model of cell cycle progression allows for heterogeneity within the cell population induced by stochastic effects. Such heterogeneous behaviour is reflected in variations in the proliferation rate. Within this set-up, we have established three main results. First, we have shown that the age to the G1/S transition, which essentially determines the birth rate, exhibits a remarkably simple scaling behaviour. Besides the fact that this simple behaviour emerges from a rather complex model, this allows for a huge simplification of our numerical methodology. A further result is the observation that heterogeneous populations undergo an internal process of quasi-neutral competition. Finally, we investigated the effects of cell-cycle-phase dependent therapies (such as radiation therapy) on heterogeneous populations. In particular, we have studied the case in which the population contains a quiescent sub-population. Our mean-field analysis and numerical simulations confirm that, if the survival fraction of the therapy is too high, rescue of the quiescent population occurs. This gives rise to emergence of resistance to therapy since the rescued population is less sensitive to therapy.

## Highlights

• Analysis and numerical simulations of a model based on an age-dependent stochastic process.
• Optimal path theory and the quasi-steady state approximation show properties related to fluctuations.
• Dynamics of a stochastic heterogeneous population under resource limitation conditions.
• Explore the effects of noise-induced heterogeneity on the emergence of drug resistance.

## 1 Introduction

Global cell traits and behaviour in response to stimuli, the so-called phenotype, results from a complex network of interactions between genes and gene products which ultimately regulates gene expression. These networks of gene regulation constitute non-linear, high-dimensional dynamical systems whose structure has been shaped up by evolution by natural selection, so that they exhibit properties such as robustness (i.e. resilience of the phenotype against genetic alterations) and canalisation (i.e. the ability for phenotypes to increase their robustness as time progresses). These properties are exploited by tumours to increase their proliferative potential and resist to therapies (Kitano, 2004). In addition to complex, non-linear interactions within individual cells, there exist intricate interactions between different components of the biological systems at all levels: from complex signalling pathways and gene regulatory networks to complex non-local effects where perturbations at whole-tissue level induce changes at the level of the intra-cellular pathways of individual cells (Alarcón et al., 2005, Ribba et al., 2006, Macklin et al., 2009, Osborne et al., 2010, Deisboeck et al., 2011, Powathil et al., 2013, Jagiella et al., 2016). These and other factors contribute towards a highly complex dynamics in biological tissues which is an emergent property of all the layers of complexity involved.

To tackle such complexity, multi-scale models of biological systems as diverse as cardiac systems (Smith et al., 2004, McCulloch, 2009, Hand and Griffith, 2010, Land et al., 2013), systems of developmental biology (Schnell et al., 2008, Oates et al., 2009, Hester et al., 2011, Setty, 2012, Walpole et al., 2013), and tumour growth systems (Alarcón et al., 2005, Jiang et al., 2005, Ribba et al., 2006, Macklin et al., 2009, Owen et al., 2009, Preziosi and Tosin, 2009, Tracqui, 2009, Byrne, 2010, Lowengrub et al., 2010, Osborne et al., 2010, Rejniak and Anderson,, Deisboeck et al., 2011, Perfahl et al., 2011, Travasso et al., 2011, Durrett, 2013, Powathil et al., 2013, Szabo and Merks, 2013, Chisholm et al., 2015, Curtius et al., 2015, Scott et al., 2016, Jagiella et al., 2016) have been developed. In parallel to the model development, algorithms and analytic methods are being developed to allow for more efficient analysis and simulation of such models (Alarcón, 2014, Spill et al., 2015, de la Cruz et al., 2015, Spill et al., 2016).

In the case of cancer biology, the multi-scale interactions of intracellular changes at the genetic or molecular pathway level and tissue-level heterogeneity can conspire to generate unfortunate effects such as resistance to therapy (Merlo et al., 2006, Gillies et al., 2012, Greaves and Maley, 2012, Chisholm et al., 2015, Asatryan and Komarova, 2016). Heterogeneity plays a major role in the emergence of drug resistance within tumours and can be of diverse types. There is heterogeneity in cell types due to increased gene mutation rate as a consequence of genomic instability and other factors (Merlo et al., 2006, Greaves and Maley, 2012, Chisholm et al., 2015, Asatryan and Komarova, 2016). Heterogeneity can also be caused by the complexity of the tumour microenvironment (Alarcón et al., 2003, Gillies et al., 2012, Chisholm et al., 2015), in which diverse factors such as tumoural or immune cells (Kalluri and Zeisberg, 2006, Grivennikov et al., 2010), or the extracellular matrix and its physical properties (Spill et al., 2016), strongly influence cancer cell behaviour. Note that hypoxia is also known to change the tumour microenvironment (Spill et al., 2016). In either case, heterogeneity within the tumour creates the necessary conditions for resistant varieties to emerge and be selected upon the administration of a given therapy.

The main aim of this paper is to analyse the properties of heterogeneous populations under the effects of fluctuations both within the intracellular pathways which regulate (individual) cell behaviour and those associated to intrinsic randomness due to finite size of the population. To this purpose, we expand upon the stochastic multi-scale methodology developed in Guerrero and Alarcón (2015), where it was shown that such a system can be described by an age-structured birth-and-death process, instead of a branching process (Danesh et al., 2012, Durrett, 2013). The coupling between intracellular and the birth-and-death dynamics is carried out through a novel method to obtain the birth rate from the stochastic cell-cycle model, based on a mean-first passage time approach. Cell proliferation is assumed to be activated when one or more of the proteins involved in the cell-cycle regulatory pathway hit a threshold. This view allows us to calculate the birth rate as a function of the age of the cell and the extracellular oxygen in terms of the associated mean-first passage time (MFPT) problem (Redner, 2001). The present approach differs from that in Guerrero and Alarcón (2015) in that our treatment of the intracellular MFPT is done in terms of a large deviations approach, the so-called optimal path theory (Freidlin and Wentzell, 1998, Bressloff and Newby, 2014).

This methodology allows us to explore the effects of intrinsic fluctuations within the intracellular dynamics, in particular a model of the oxygen-regulated G1/S which dictates when cells are prepared to divide, as a source of heterogeneous behaviour: fluctuations induce variability in the birth rate within the population (even to the point of rendering some cells quiescent, i.e. stuck in G0) upon which a cell-cycle dependent therapy acts as a selective pressure.

This paper is organised as follows. Section 2 provides a summary of the structure of the multi-scale. In Section 3, we give a detailed discussion of the intracellular dynamics, i.e. the stochastic model of the oxygen-regulated G1/S transition, and its analysis. In Section 4, we summarise the formulation of the age-structured birth-and-death process, the numerical simulation technique, and the mean-field analysis of a homogeneous population. In Section 5, we discuss how noise within the intracellular dynamics induces heterogeneity in the population and analyse the stochastic dynamics of competition for a limited resource within such heterogeneous populations. In Section 6 we further study the effects of noise-induced heterogeneity on the emergence of drug resistance upon administration of a cell cycle-specific therapy. Finally, in Section 7 we summarise our results and discuss our conclusions as well as avenues for future research.

## 2 Summary of the multi-scale model

Before proceeding with a detailed discussion of the different elements involved in the formulation of the stochastic multi-scale model, it is useful to provide a general overview of the overall structure of the model, which is closely related to that of the model proposed in Alarcón et al. (2005).

**Fig. 1:**
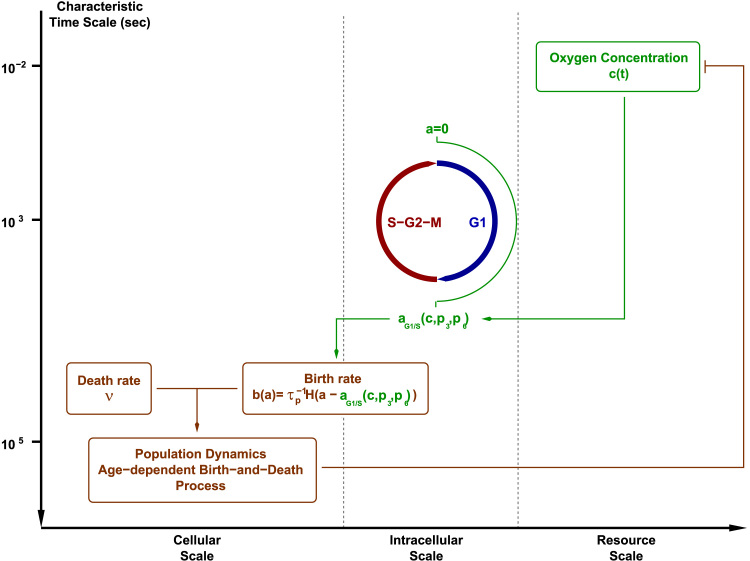
Schematic representation of the different elements that compose our multi-scale model. We show the different levels of biological organisation as well as associated characteristic time scales (Guerrero and Alarcón, 2015) associated to each of these layers: resource scale, i.e. oxygen which is supplied at a constant rate and consumed by the cell population, cellular scale, i.e. oxygen-regulated cell cycle progression which determines the age-dependent birth rate into the cellular layer, and, finally, the cellular scale, which is associated to the stochastic population dynamics.

The model we present in this article integrates phenomena characterised by different time scales, as schematically shown in Fig. 1. This model intends to tackle the growth and competition of cellular populations under the restriction of finite amount of available resources (in this case, oxygen) supplied at a finite rate, $\overset{¯}{S}$.

The general approach used in this model is a natural generalisation of the standard continuous-time birth-and-death Markov process and its description via a Master Equation (Gardiner, 2009). As we will see, the consideration of the multi-scale character of the system, i.e. the inclusion of the physiological structure associated with the cell-cycle variables, introduce an age-structure within the population: the birth rate depends on the age of cell (i.e. time elapsed since last division) which determines, through the corresponding cell-cycle model, the cell-cycle status of the corresponding cells.

The evolution of the concentration of oxygen, *c*(*t*), (*resource scale*, see Fig. 1) is modelled by:

(1) $$\frac{\mathrm{dc}}{\mathrm{dt}}=\overset{¯}{S}-\overset{¯}{k}c\overset{N_{T}}{\underset{i=1}{\sum }}N_{i}(t)$$

where *N*_*T*_ is the number of cellular types consuming the resource *c*, and *N*_*i*_(*t*), $i=1,\ldots ,N_{T}$, is the number of cells of type *i* at time *t*. Note that, in general, *N*_*i*_(*t*) is a stochastic process, and, therefore, in principle Eq. (1) should be treated as a stochastic differential equation (Oksendal, 2003).

The second sub-model considered in our multi-scale model, associated with the intracellular scale (see Fig. 1), is a stochastic model of oxygen-regulated cell-cycle progression. This sub-model is formulated using the standard techniques of chemical kinetics modelling (Gillespie, 1976) so that the mean-field limit of the stochastic model corresponds to the deterministic cell-cycle model formulated in Bedessem and Stephanou (2014). This model provides the physiological state of the cell in terms of the number of molecules of each protein involved in the cell-cycle of a cell of a given age, *a*. From such a physiological state, we derive whether the G1/S transition has occurred. The cell-cycle status of a cell of age *a* is determined in terms of whether the abundance of certain proteins which activate the cell-cycle (cyclins) have reached a certain threshold. In our particular case, if at age *a*, the cyclin levels are below the corresponding threshold, the cell is still in G1. If, on the contrary, the prescribed threshold level has been reached, the cell has passed onto S, and therefore is ready to divide. This implies that the probability of a cell having crossed the threshold of cyclin levels at age *a* can be formulated in terms of a mean first-passage time problem (MFTP) in which one analyses the probability of a Markov process to hit a certain boundary (Redner, 2001, Gardiner, 2009). Unlike our approach in Guerrero and Alarcón (2015), based on approximating the full probability distribution of the stochastic cell cycle model, in the present approach, passage time is (approximately) solved in terms of an optimal trajectory path approach (Freidlin and Wentzell, 1998, Bressloff and Newby, 2014).

At the interface between the intracellular and cellular scales sits our model of the (age-dependent) birth rate, which defines the probability of birth per unit time (cellular scale) in terms of the cell cycle variables (intracellular scale). The rate at which our cell-cycle model hits the cyclin activation threshold, i.e. the rate at which cells undergo G1/S transition, is taken as proportional to the birth rate. In particular, the birth rate is taken to be a function of the age of the cell as well as the concentration of oxygen, as the oxygen abundance regulates the G1/S transition age, $a_{G1/S}(c)$, i.e. the time (age) elapsed between the birth of a cell and its G1/S transition:

(2) $$b(a)=\tau_{p}^{-1}H(a-a_{G1/S}(c)).$$

i.e. cell division occurs at a constant rate, $\tau_{p}^{-1}$, provided cells undergo the G1/S transition and *H* is the Heaviside function. In other words, we consider that the duration of the G1 phase is regulated by the cell cycle model, whereas the duration of the S-G2-M is a random variable, exponentially distributed with average duration equal to *τ*_*p*_ (see Fig. 1).

The third and last sub-model is that associated with the cellular scale. It corresponds to the dynamics of the cell population and is governed by the Master Equation for the probability density function of the number of cells (Gardiner, 2009). The stochastic process that describes the dynamics of the population of cells is an age-dependent birth-and-death process where the birth rate is given by Eq. (2) where $a_{G1/S}(c)$ is provided by the intracellular model. The death rate is, for simplicity, considered constant. As a consequence of the fact that the birth rate is age-dependent, our Multi-Scale Master Equation (MSME) does not present the standard form for unstructured populations, rather, it is an age-dependent Master Equation.

A detailed description of each sub-model and its analysis is given in 3, 4.

## 3 Intracellular scale: stochastic model of the oxygen-regulated G_1_/S transition

### 3.1 Biological background

Cell proliferation is orchestrated by a complex network of protein and gene expression regulation, the so-called cell cycle, which accounts for the timely coordination of proliferation with growth and, by means of signalling cues such as growth factors, tissue function (Yao, 2014). Dysregulation of such an orderly organisation of cell proliferation is one of the main contributors to the aberrant behaviour observed in tumours (Weinberg, 2007).

The cell cycle has the purpose of regulating the successive activation of the so-called cyclin-dependent kinases (CDKs) which control the progression along the four phases of cycle: G1 (first gap phase), S (DNA replication), G2 (second gap phase), and M (mitosis) (Gerard and Goldbeter, 2009, Gerard and Goldbeter, 2011, Gerard et al., 2015). These four phases must be supplemented with a fifth, G0, which accounts for cells that are quiescent due to lack of stimulation (i.e. absence of growth factors, lack of basic nutrients, etc.) to proliferate. Recent models of the cell cycle organise the complex regulatory network into CDK modules, each centred around a cyclin–CDK complex which is key for the transition between the cell cycle phases (see, for example, Gerard and Goldbeter, 2009): cyclin D/CDK4-6 and cyclin E/CDK2 regulate progression during the G1 phase and elicit the G1/S transition, cyclin A/CDK2 promote progression during S phase and orchestrates the S/G2 transition, and, finally, cyclin B/CDK2 brings about the G2/M transition. The activity of each of these cyclin-CDK complexes is regulated in a timely manner, so that each phase of the cell cycle ensues at the proper time, by means of transcriptional regulation, post-transcriptional modifications (e.g. phosphorylation), and degradation (via ubiquitination) in which a large number of other components participate, including transcription factors, enzymes, ubiquitins, etc.

In the present paper, we propose a coarse-grained description of the cell cycle phases by lumping S, G2, and M into one phase, so that we consider a two-phase model G1 and S-G2-M, as shown in Fig. 1, Alarcón et al. (2004). In particular, we consider that cells can only divide once they have entered the S-G2-M phase at a constant rate. Entry in S-G2-M is regulated by a (stochastic) model of the G1/S transition which takes into account the regulation of the duration of the G1 phase by hypoxia (lack of oxygen).

**Fig. 2:**
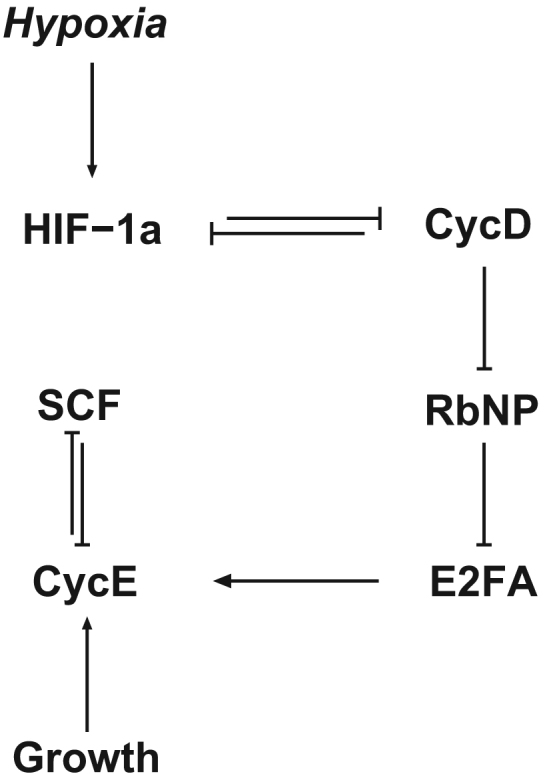
Schematic representation of the elements involved in the model of hypoxia-regulated G_1_/S transition proposed by Bedessem and Stephanou (2014). Within the framework of this model, the negative-feedback between CycE and SCF is the key modelling ingredient for the system to emulate the behaviour of a cell during the G1/S transition. The relative balance between CycE (which promotes the G1/S transition) and SCF (G1/S transition inhibitor) is regulated by growth and hypoxia, so that the timing of the transition depends upon these two factors.

The abundance of oxygen is known to be one of the factors that regulates the duration of the G_1_ phase of the cell cycle. The issue of the regulation of the G_1_/S transition by the oxygen concentration has been the subject of several modelling studies (Alarcón et al., 2004, Bedessem and Stephanou, 2014). These models focus on the hypoxia-induced delay of the activation of the cyclins either through activation of cyclin inhibitors (Alarcón et al., 2004) or via up-regulation of the HIF-1*α* transcription factor (Bedessem and Stephanou, 2014). From the modelling point of view, both of them are mean-field models, thus neglecting fluctuations. In this section, we formulate a stochastic version of the model of Bedessem and Stephanou (2014), of which a schematic representation is shown in Fig. 2.

HIF-1 mediates adaptive responses to lack of oxygen (Semenza, 2013). HIF-1 is a heterodimer consisting of two sub-units: HIF-1*α* and HIF-1*β*. Whilst the latter is constitutively expressed, HIF-1*α* is O_2_-regulated. In the presence of adequate oxygen availability is negatively regulated by the von Hippel–Lindau (VHL) tumour suppressor protein, which allows HIF-1*α* for degradation. VHL loss-of-function mutations are common in many types of tumours, which allows for de-regulated HIF-1*α* degradation (Semenza, 2013). HIF-1 is involved in a number of cellular responses including switch from oxidative phosphorylation to glycolysis, activation of angiogenic pathways, and inhibition of cell cycle progression (Bristow and Hill, 2008, Semenza, 2013).

### 3.2 Mean-field model of the regulation of the G1/S transition by hypoxia

Bedessem and Stephanou formulate a model of the effect of hypoxia (mediated by HIF-1) on the timing of the G1/S transition (Bedessem and Stephanou, 2014). The involvement of HIF-1 in cell cycle regulation is complex and not completely understood. There is evidence that HIF-1 delays entry into S(-G2-M) phase by activating p21, a CDK inhibitor (Koshiji et al., 2004, Ortmann et al., 2014). HIF-1 up-regulation of p21 mediates indirect down-regulation of cyclin E (Goda, 2003). Further to HIF-1 regulation of the cell cycle, there exists a feedback regulation of cell cycle proteins of HIF-1. Hubbia et al. (2014) report that CDK1 and CDK2 physically and functionally interact with HIF-1*α*: CDK1 down-regulates lysosomal degradation of HIF-1*α*, thus consolidating its stability and promoting its transcriptional activity. On the contrary, CDK2 activates lysosomal degradation of HIF-1*α* and promotes G1/S transition. Bedessem and Stephanou (2014) do not take into account all these issues and, for simplicity, chose to focus on the well-documented effect of HIF-1 on cyclin D (Wen et al., 2010, Ortmann et al., 2014).

Bedessem and Stephanou (2014) model formulation is based on the following assumptions:

1. The G1/S is modelled by a biological switch which emerges from the mutual inhibition between a cyclin (in this case, cyclin E) and a ubiquitin complex (SCF complex): The latter marks the former for degradation whereas cyclin E mediates inactivation of the SCF complex. This mutual inhibition gives rise to a bistable situation in which two stable fixed points coexist, each associated with the G1 phase (high SCF activity, low cyclin E concentration) and the S-G2-M phase (low SCF activity, high cyclin E concentration). Activation and inactivation of the SCF complex are assumed to follow Michaelis–Menten kinetics.
2. As in previous models (Tyson and Novak, 2001, Novak and Tyson, 2004, Alarcón et al., 2004), the G1/S transition is brought about by triggering a saddle-node bifurcation, whereby the G1 phase fixed point collides with the unstable saddle, driving the system into S-G2-M fixed point. Two factors drive the system through this bifurcation: cell growth (logistic increase of the cell mass, Tyson and Novak, 2001) and activation of the E2F transcription factor. In Bedessem and Stephanou (2014), both factors are taken to up-regulate the transcription of cyclin E.
3. Activation of E2F is modelled in terms of the E2F-Retinoblastoma protein (RBP) switch (Lee et al., 2010). Briefly, E2F is captured (bound) by unphosphorylated RBP. Upon phosphorylation, RBP releases E2F which activates transcription of G1/S-transition promoting cyclins, such as cyclin E (Alberts et al., 2002). Further, E2F can be in unphosphorylated (active) form and phosphorylated (inactive) form. Following Novak and Tyson (2004), Bedessem and Stephanou assume that fraction of active E2F and RBP-bound E2F are in adiabatic equilibrium with unphosphorylated RBP and total free E2F (Bedessem and Stephanou, 2014). Furthermore, (Bedessem and Stephanou (2014)) takes cyclin D to phosphorylate RBP.
4. Last, Bedessem and Stephanou (2014) assume that HIF-1*α* inhibits synthesis of cyclin D. Following experimental evidence reported in Wen et al. (2010), they assume that the level of HIF-1*α* depends exponentially of the oxygen concentration.

These basic hypotheses are primarily based on previous models (Tyson and Novak, 2001, Novak and Tyson, 2004, Alarcón et al., 2004). The resulting pathway is schematically represented in Fig. 2.

### 3.3 Stochastic G1/S transition model

**Fig. 3:**
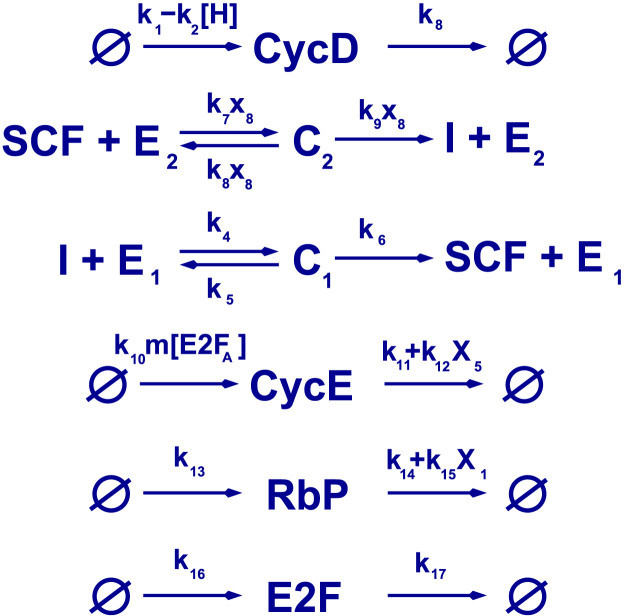
Reactions for the stochastic version of the model proposed by Bedessem and Stephanou (2014). The reactions correspond (from top to bottom) to: hypoxia-inhibited synthesis and degradation of CycD, enzyme-catalysed, CycE-mediated inactivation of SCF, enzyme-catalysed activation of SCF, synthesis (regulated by growth and active E2F) and degradation (up-regulated by active SCF) of CycE, synthesis and degradation of Rb, and, last, synthesis and degradation of E2F. The negative feedback (mutual inhibition) between SCF and CycE mediates bistable behaviour in this model of the G1/S transition. Some of the transition rates associated to the reactions shown in are not constant but depend on the number of molecules of chemical species *i* present at a particular time, *X*_*i*_. For the definition of the quantities *X*_*i*_, we refer the reader to Table 1.

Based on the hypotheses summarised in Section 3.2, Bedessem and Stephanou (2014) formulated a mean-field model which is able to reproduce such behaviours as delay of the G1/S due lo lack of oxygen as well as hypoxia-induced quiescence. Here, we present a stochastic model (see Fig. 3 and Table 1), whose mean field limit is the model formulated in Bedessem and Stephanou (2014). We analyse this model using the stochastic quasi-steady state approximation we have developed in Alarcón, 2014, de la Cruz et al., 2015.

The deterministic model formulated in Bedessem and Stephanou (2014), which we have briefly described in Section 3.2, is based on a series of reactions shown in Fig. 3, which include Michaelis–Menten kinetics for activation and inactivation of SCF complexes. Our stochastic model of the G1/S transition builds upon the stochastic (Markovian) description of the same set of reactions.

Our model is predicated on the stochastic dynamics of the state vector being described by a Markov jump process (Grimmett and Stirzaker, 2001, Kampen, 2007), whereby the state of the system, *X*, changes by an amount *r*_*i*_ when the elementary reaction *i* occurs. The waiting time between Markovian events is exponentially distributed, the process is characterised by the associated transition rates, i.e. $P(X(a+\Delta a)=X+r_{i}|X(a)=X)=W_{i}(X)\Delta a+O(\Delta a^{2})$. Using law of mass action (Gillespie, 1976) as our basic modelling framework, the transition rates of each elementary process are given in Table 1. Once we have determined the transition rates associated with each elementary reaction (or channel), the dynamics of the system is given by the Chemical Master Equation of a (non-structured) Markov Process, X(a):

(3) $$\frac{\partial P(X,a)}{\partial a}=\overset{R}{\underset{i=1}{\sum }}\left(W_{i}(X-r_{i})P(X-r_{i},a)-W_{i}(X)P(X,a)\right)$$

where P(X,a) is the probability of the state vector of the system to be *X* at age, i.e. the time reckoned from the last division, *a*. The transition rates, $W_{i}(X)$, the vectors *r*_*i*_ (whose components are the variation of the number of each chemical species upon occurrence of reaction *i*) are given in Table 1 and determine the dynamics of the system.

Even for moderately complex models, Eq. (3) has no solution in closed form. Therefore, in order to study the properties of the system one must resort to numerical simulation (Monte Carlo) or asymptotic approximations. In the next section, we present an asymptotic analysis based on a recently developed form of stochastic quasi-steady state approximation.

### 3.4 Semi-classical quasi-steady state analysis of the stochastic G1/S transition model

In Alarcón, 2014, de la Cruz et al., 2015, Sanchez-Taltavull,, we have developed a stochastic version of the classical QSS approximation, the so-called semi-classical QSS approximation (SCQSSA) which, within the framework of the optimal path theory, allows us to tackle systems which exhibit separation of time scales, such as enzyme-catalysed reactions. Since these types of reaction feature prominently in our stochastic model of the hypoxia-regulated G1/S transition (see Fig. 3), we will use the SCQSSA to analyse the effects of intrinsic noise on the stochastic model of the hypoxia-regulated G1/S transition (as determined by the transition rates shown in Table 1). This approximation allows us to study noise-induced phenomena which are relevant for the timing of the G1/S transition and, therefore, bear upon the population dynamics.

Following the SCQSSA methodology summarised in Appendix A, we derive the following set of equations which describe the optimal path associated with the stochastic G1/S transition model, Table 1:

(4) $$\frac{{\mathrm{dq}}_{1}}{\mathrm{da}}=\kappa_{1}-\kappa_{2}-\kappa_{3}q_{1}$$

(5) $$\frac{{\mathrm{dq}}_{5}}{\mathrm{da}}=\frac{\kappa_{6}p_{3}p_{e_{1}}(p_{c}-q_{5})}{(p_{c}-q_{5})+\frac{\kappa_{5}+\kappa_{6}}{\kappa_{4}}}-\frac{\kappa_{9}p_{6}p_{e_{2}}q_{5}q_{8}}{q_{5}+(\kappa_{8}+\kappa_{9})}$$

(6) dq8da=κ10mq10(1−q9[e2f]^tot)−κ11q8−κ12q5q8

(7) $$\frac{{\mathrm{dq}}_{9}}{\mathrm{da}}=\kappa_{13}-\kappa_{14}q_{9}-\kappa_{15}q_{1}q_{9}$$

(8) $$\frac{{\mathrm{dq}}_{10}}{\mathrm{da}}=\kappa_{16}-\kappa_{17}q_{10}$$

(9) $$q_{2}=p_{c}-q_{5}$$

(10) $$q_{4}=\frac{p_{e_{1}}q_{2}}{q_{2}+\frac{\kappa_{5}+\kappa_{6}}{\kappa_{4}}},\;q_{3}+q_{4}=p_{e_{1}}$$

(11) $$q_{7}=\frac{p_{e_{2}}q_{5}}{q_{5}+(\kappa_{8}+\kappa_{9})},\;q_{6}+q_{7}=p_{e_{2}}$$

(12) $$p_{3}=\mathrm{cnt}.\;p_{6}=\mathrm{cnt}.$$

where *a* is the rescaled age variable $a=k_{7}\mathrm{ESt}$ (see Appendix B, Table B1). The oxygen dependencies enter the model though the [H] dependent parameter *κ*_2_ (see Table B1, Table B2, Table B3). The reader is referred to Appendix A for a summary of the method and Appendix B for a detailed derivation of Eqs. (4), (B.36), (B.37), (7), (8), (9), (10), (11), (12) which hereafter is referred to as the semi-classical quasi-steady state approximation (SCQSSA) of the stochastic cell-cycle model and to Alarcón, 2014, de la Cruz et al., 2015 for a full account of the SCQSSA methodology. Note that there are other approximations which do not require the QSSA assumption, such as the one described in Ball et al., 2006, Kurtz, 1978. Furthermore, since we will be interested in the random effects associated to the enzyme-regulated dynamics of SCF, encapsulated in the parameters *p*_3_ and *p*_6_, we have taken $p_{i}(a)=1$ for all i≠3,6. This corresponds to analysing the marginal distribution integrating out all the stochastic effects associated to all *X*_*i*_ with i≠3,6.

### 3.5 Stochastic behaviour of the G1/S model

We now proceed to study the behaviour of the stochastic model of the oxygen-regulated G1/S transition. We pay special attention to those aspects in which we observe a departure of the stochastic system from the mean-field behaviour. In particular, we highlight the effects of modifying the relative abundance of SCF-activating and inactivating enzymes, including the ability of inducing oxygen-independent quiescence.

#### 3.5.1 The relative abundance of the SCF activating and inactivating enzymes controls the timing of the G1/S transition

In references Alarcón, 2014, de la Cruz et al., 2015, we have shown that, under SCQSSA conditions, the momenta *p*_3_ and *p*_6_, i.e. the momenta coordinates associated with the SCF-activating and inactivating enzymes, respectively, are determined by the probability distribution of their initial (conserved) number. In particular, if we assume that the initial number of SCF-activating and inactivating enzyme molecules, *E*_1_ and *E*_2_, is distributed over a population of cells following a Poisson distribution with parameter *E*, we have shown that (de la Cruz et al., 2015):

$$p_{3}=\frac{e_{1}}{E}=p_{e_{1}},\;p_{6}=\frac{e_{2}}{E}=p_{e_{2}}.$$

**Fig. 4:**
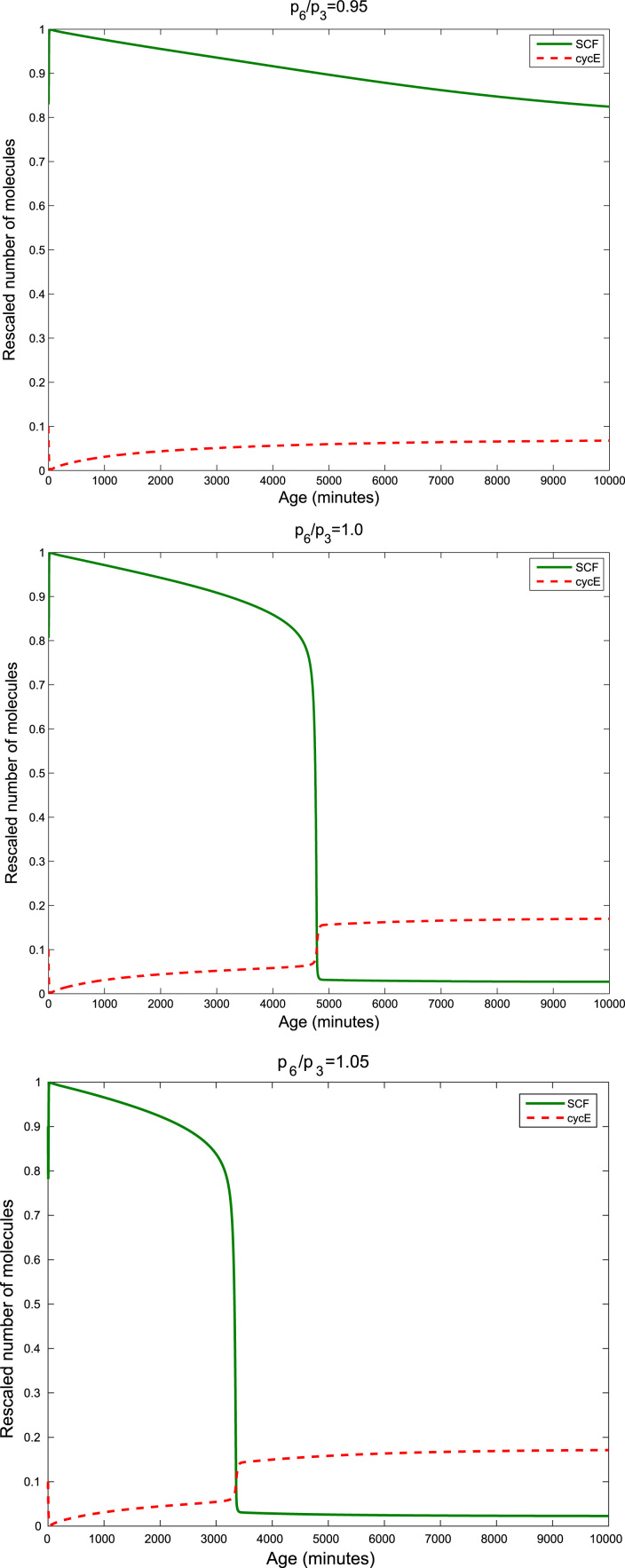
Series of plots illustrating how the ratio $p_{6}/p_{3}$, which is associated with the ratio of the number of SCF-inactivating and SCF-activating enzymes, modulates the timing of the G1/S transition. Parameter values as given in Table B2. Initial conditions are provided in Table B4.

**Fig. 5:**
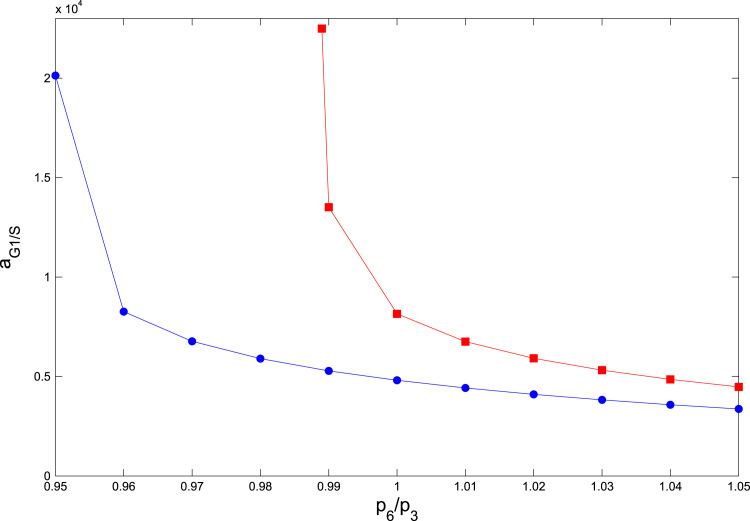
Plot showing how the G1/S transition age, $a_{G1/S}(c,p_{3},p_{6})$, changes as the ratio $p_{3}/p_{6}$, which is determined by the ratio of the (conserved) amounts of SCF activating and inactivating enzymes (Alarcón, 2014, de la Cruz et al., 2015), varies. We show $a_{G1/S}(c,p_{3},p_{6})$ for *c*=1 (blue circles) and *c*=0.1 (red squares). Parameter values as given in Table B2. (For interpretation of the references to color in this figure caption, the reader is referred to the web version of this paper.)

With this in mind, we can analyse the effect of changing the relative concentration of SCF-activating and inactivating enzymes on the timing of the G1/S transition. Our results are shown in Fig. 4, Fig. 5. Fig. 4 illustrates that, for a fixed oxygen concentration, the G1/S transition is delayed by depriving the system of SCF-activating enzyme: as the ratio $p_{3}/p_{6}=e_{1}/e_{2}$ of SCF activating and deactivating enzyme increases, the G1/S transition takes longer to occur. Then, Fig. 5 shows that the G1/S transition age $a_{G1/S}(c,p_{3},p_{6})$ decreases when $p_{3}/p_{6}$ increases. Furthermore, increasing the oxygen concentration *c* from *c*=0.1 to *c*=1 shifts the curve towards lower transition ages $a_{G1/S}(c,p_{3},p_{6})$. Note that this prediction is beyond the reach of the mean-field limit Bedessem and Stephanou (2014).

#### 3.5.2 Induction of quiescence

**Fig. 6:**
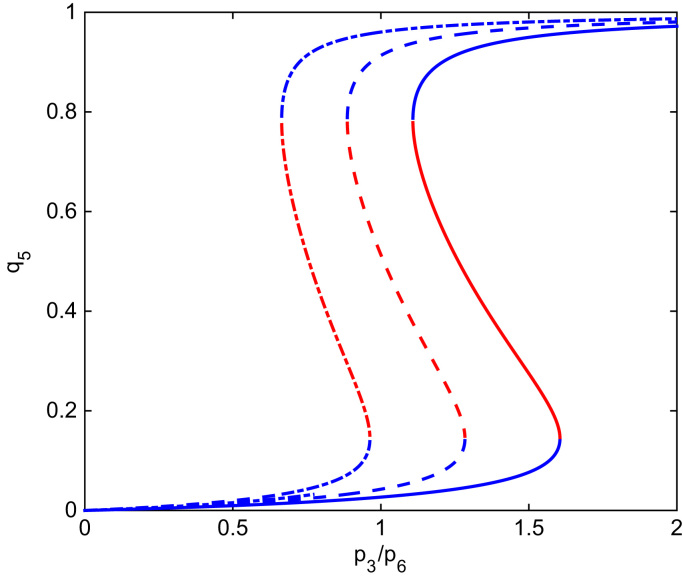
This figure shows the bifurcation diagram of the SCQSSA of the stochastic cell-cycle model for different values of the parameter *m*. The ratio $p_{3}/p_{6}$ is the control parameter. The order parameter is the steady state value of the generalised coordinate associated with active SCF, *q*_5_. Solid line corresponds to *m*=10, dash lines to *m*=8 and dotted lines to *m*=6. Parameter values as give in Table B3, $p_{i}=1,\;i\neq 3,6$, *p*_*c*_=1 and *c*=1. Blue lines indicate stable steady state and red lines indicate unstable steady state. (For interpretation of the references to color in this figure caption, the reader is referred to the web version of this paper.)

In view of the results of Section 3.5.1, we have proceeded to a more thorough analysis of the effect of varying the ratio $p_{3}/p_{6}$, which we recall that, within the SCQSSA, is equal to the ratio between the abundance of SCF-activating and inactivating enzymes, on the behaviour of the SCQSSA system Eqs. (4), (B.36), (B.37), (7), (8), (9), (10), (11), (12). In particular, we have investigated the bifurcation diagram of Eqs. (4), (B.36), (B.37), (7), (8), (9), (10), (11), (12) with $p_{3}/p_{6}$ as the control parameter. Our results are shown in Fig. 6. We observe that, regardless of the value of *m*, there exists a range of values of the control parameter for which the saddle-node bifurcation, which gives rise to the G1/S transition, does not occur (i.e. only the G1-fixed point is stable). This result implies that depletion of SCF-activating enzyme, or, equivalently, over-expression of SCF-inactivating enzyme can stop cell-cycle progression by locking cells into the so-called G0 state, i.e. quiescence.

**Fig. 7:**
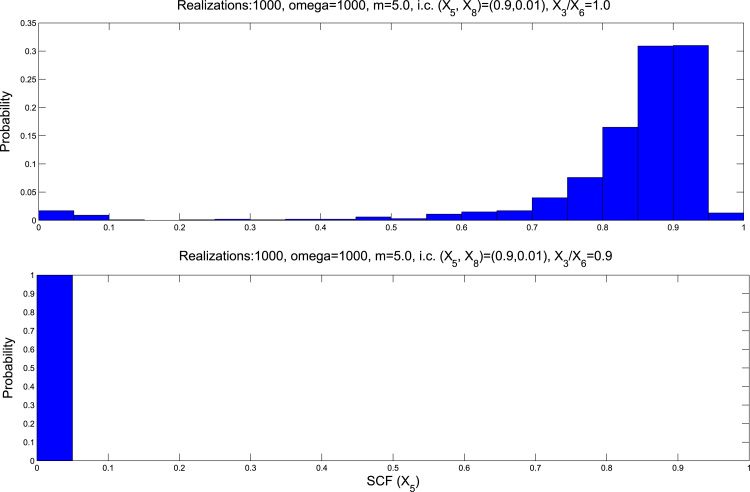
Simulation results for the stochastic model of the oxygen-regulated G1/S transition defined by the transition rates given in Table 1. We have plotted the probability $P(X_{5},T)$, where *T*=100, with different values of $X_{3}(\tau =0)/X_{6}(\tau =0)$. 1000 realisations and *m*=5.0.

These results are confirmed by direct simulation of the stochastic cell-cycle model (Table 1) using Gillespie's stochastic simulation algorithm (Gillespie, 1976), see Fig. 7.

### 3.6 Scaling theory of the G1/S transition age

We finish our analysis of the intracellular dynamics by formulating a scaling theory of one of the fundamental quantities in our multi-scale model, namely, the G1/S transition age, $a_{G1/S}(c,p_{6},p_{3})$, which determines the age-dependent birth rate (see Eq. (2)). In this section, we will show that, in spite of the complexity of the SCQSSA formulation of the oxygen-dependent cell-cycle progression model (see Eqs. (4), (B.36), (B.37), (7), (8), (9), (10), (11), (12)), $a_{G1/S}$ exhibits remarkable regularities with respect to its dependence on the oxygen concentration and the cell-cycle parameters *p*_6_ and *p*_3_. Such regularities hugely simplify our multi-scale methodology.

**Fig. 8:**
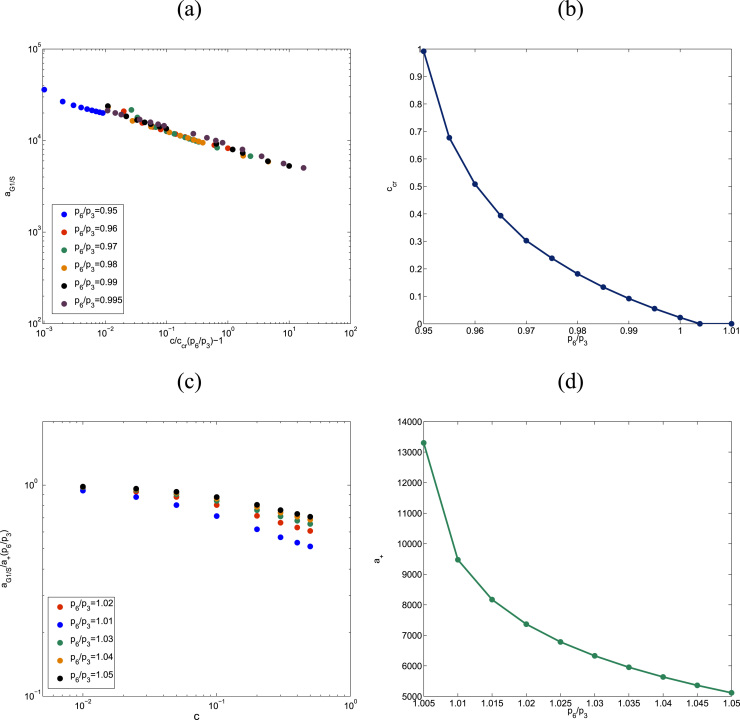
Series of plots showing the scaling analysis of the G1/S transition age. Plot (a) shows that below the critical value *r*_*cr*_, which corresponds to the value of the ratio $p_{6}/p_{3}$, above which there is no transition to quiescence (i.e. if $p_{6}/p_{3}>r_{\mathrm{cr}}$ the G1/S transition age is finite when *c*=0), $a_{G1/S}(c,p_{6},p_{3})$ follows an algebraic decay with a universal $p_{6}/p_{3}$-independent exponent provided that the oxygen, *c*, is rescaled by the critical oxygen concentration, $c_{\mathrm{cr}}(p_{6}/p_{3})$. Plot (c) shows that if, by contrast, $p_{6}/p_{3}>r_{\mathrm{cr}}$$a_{G1/S}(c,p_{6},p_{3})$ decays exponentially with the oxygen concentration with a characteristic concentration *c*_0_ which, provided $p_{6}/p_{3}$ is larger enough than $r_{\mathrm{cr}},$ is $p_{6}/p_{3}$-independent. Plot (b) shows how *c*_*cr*_ varies as $p_{6}/p_{3}$ is changed. Similarly, plot (d) shows how $a_{+}$ varies $p_{6}/p_{3}$ changes. Parameter values as given in Table B2.

We can see this regularity in Fig. 8. Fig. 8(a) shows $a_{G1/S}$ as a function of the ratio $\left(\frac{c}{c_{\mathrm{cr}}(p_{6}/p_{3})}-1\right)$, for six different values of $\frac{p_{6}}{p_{3}}<r_{\mathrm{cr}}$, where $r_{\mathrm{cr}}≃1.004$ is the critical value. We see that all graphs fall together on a straight line in log–log space, indicating a power-law dependence of $a_{G1/S}$ on $\left(\frac{c}{c_{\mathrm{cr}}(p_{6}/p_{3})}-1\right)$. On the other hand, Fig. 8(c) shows the dependence of the normalized function $\frac{a_{G1/S}(c,\frac{p_{6}}{p_{3}})}{a_{+}(p_{6}/p_{3})}$, on *c*, for five values $\frac{p_{6}}{p_{3}}>r_{\mathrm{cr}}$. Here, $a_{+}(p_{6}/p_{3})=a_{G1/S}(c=0,p_{6}/p_{3})$. We see that there is good agreement between the five values $\frac{p_{6}}{p_{3}}$ for small *c*, with the disagreement increasing with increasing *c*. Thus, a good scaling approximation for $a_{G1/S}$ is given by

(13) $$a_{G1/S}\left(c,\frac{p_{6}}{p_{3}}\right)≃\{\begin{array}{c} a_{+}(\frac{p_{6}}{p_{3}})e^{-c/c_{0}}\;\mathrm{if}\;\frac{p_{6}}{p_{3}}>r_{\mathrm{cr}}\\ a_{-}{\left(\frac{c}{c_{\mathrm{cr}}(p_{6}/p_{3})}-1\right)}^{-\beta }\;\mathrm{if}\;\frac{p_{6}}{p_{3}}<r_{\mathrm{cr}} \end{array}$$

Here, *c*_0_, $a_{-}$, and *β* are constants. According to our analysis of the data presented in Fig. 8, these constants are given by $c_{0}≃1.1$, $a_{-}≃8.25\cdot 10^{3}$ and β≃0.2. Likewise, $a_{+}(p_{6}/p_{3})$ is obtained by fitting to the data presented in Fig. 8(d). The critical oxygen concentration for quiescence *c*_*cr*_ can be estimated analytically (with parameter values taken from Table B2):

(14) $$c_{\mathrm{cr}}\left(\frac{p_{6}}{p_{3}}\right)=1-\frac{1}{\beta_{1}}\log(\frac{1}{a_{3}H_{0}}(a_{1}+\frac{a_{2}d_{2}}{d_{1}{\left[e2f\right]}_{t}}(1-\frac{1}{1-a_{0}{\left(\frac{p_{3}}{p_{6}}\right)}^{2}})))$$

where *a*_1_, *a*_3_, *d*_1_, *d*_2_, ${\left[e2f\right]}_{t}$, and *H*_0_ are parameters defined in Appendix B, Table B2. The parameter *a*_0_ can be estimated as follows. Let *A*(*c*) be defined as:

(15) $$A(c)={\left(\frac{p_{6}}{p_{3}}\right)}^{2}(1-\frac{d_{2}}{(d_{2}+d_{1}\frac{a_{1}-a_{3}[H]}{a_{2}}){\left[e2f\right]}_{t}})$$

where $[H]=a_{3}H_{0}e^{\beta_{1}(1-c)}$. $a_{0}=A(c_{\mathrm{bif}})$, where *c*_*bif*_ is the critical value of the oxygen concentration for which the saddle node bifurcation occurs, i.e. the critical value for which the number of real, positive solutions of the equation:

(16) $$e_{1}(1-x)(J_{4}+x)(b_{2}+b_{3}x)=A(c)e_{2}b_{1}m_{*}{\left[e2f\right]}_{t}x(1+J_{3}-x)$$

goes from 3 to 1. The parameters *b*_*i*_, *e*_*i*_, and *J*_*i*_ are defined in Appendix B, Table B2

## 4 Cellular scale: multi-scale Master Equation and path integral formulation

We start by summarising the formulation of the multi-scale Master Equation (MSME) for the population dynamics model in terms of an age-structured stochastic process (Guerrero and Alarcón, 2015). First, we consider a simple age-dependent birth-and-death process where n(a,t) stands for the number of cells of age *a* at time *t*. Both *a* and *t* are dimensionless according to the scaling prescribed in Table B1, Appendix B. The time variable is $t\to k_{7}\mathrm{ESt}$ and *a* is defined after Eq. (12). The offspring of such cells with age *a*=0.

(17) P(n,a+δa,t+δt)=W(n+1,a,t)δtP(n+1,a,t)+(1−W(n,a,t)δt)P(n,a,t),

where W(n(a),a,t)=(ν+b(a))n(a) (see Table 2), *μ* is the (age-independent) death rate, and the age-dependent birth rate, *b*(*a*), is given by

(18) $$b(a)=\{\begin{array}{c} 0\;\mathrm{if}\;q_{8}(a)<{\mathrm{CycE}}_{T}\\ \tau_{p}^{-1}\;\mathrm{if}\;q_{8}(a)\geq {\mathrm{CycE}}_{T} \end{array}$$

where $q_{8}(a)$ is the generalised coordinate associated with the concentration of CycE which must exceed a threshold value, CycE_*T*_ for the cell-cycle to progress beyond the G_1_/S transition. Before this transition occurs, cells are not allowed to divide.

By re-arranging Eq. (17) and taking the limit δt=δa→0, we obtain:

(19) $$\frac{\partial P(n,a,t)}{\partial t}+\frac{\partial P(n,a,t)}{\partial a}=W(n+1,a,t)P(n+1,a,t)\;-W(n,a,t)P(n(a),a,t).$$

where $W(n,a,t)=W_{1}(n,a,t)+W_{2}(n,a,t)$ (see Table 2). Eqs. (19) needs to be supplemented with the appropriate boundary condition at *a*=0, $P(n_{0},a=0,t)$. We proceed by first considering the number of births that occur within the age group $a=a_{j}$ during a time interval of length δt. Since we are assuming that our stochastic model is a Markov process where, within each age group, birth and death occur independently and with exponentially distributed waiting times, the number of births, $B(a_{j})$, is distributed according to a Poisson distribution:

$$P(B(a_{j})=b_{j}|\delta t)=e^{-b(a_{j})n(a_{j},t)\delta t}\frac{{\left(b(a_{j})n(a_{j},t)\delta t\right)}^{b_{j}}}{b!}.$$

The total number of births delivered by the whole population during δt, *B*, is $B=\sum_{i\in I(t)}B(a_{j})$. Its probability density is therefore given by:

(20) $$P(B=b_{0})=\underset{\left\{b_{j}\right\}}{\sum }(\underset{j}{\prod }P_{j}(B(a_{j})=b_{j}))\delta_{\underset{j\in I(t)}{\sum }b_{j},b_{0}}.$$

Since Eq. (20) is a convolution, using the well-known property of the probability generating function, the generating function associated with *P*(*B*), $G_{B}(p,t)$ is given by Grimmett and Stirzaker (2001):

$$G_{B}(p,t)=\underset{j\in I(t)}{\prod }G_{j}(p,t),$$

where $G_{j}(p,t)$ is the generating function associated with $P_{j}(B(a_{j}))$:

$$G_{j}(p,t)=e^{b(a_{j})n(a_{j})\delta t(p-1)}.$$

Therefore, by taking δt=δa→0

(21) $$G_{B}(p,t)=e^{(p-1)(\underset{a}{\sum }b(a)n(a))\delta t}.$$

Eq. (21) determines $P(n_{0},a=0,t)$ since $P(n_{0},a=0,t)=P(B=n_{0}/2,t)$ and

(22) $$P(B=b_{0},t)=\frac{1}{b_{0}!}{\frac{\partial^{b_{0}}G_{B}}{\partial p^{b_{0}}}|}_{p=0}.$$

### 4.1 Numerical method

Before proceeding further, we briefly describe the numerical methodology that we use to simulate the stochastic multi-scale model. In essence, the numerical method is an extension of the hybrid stochastic simulation algorithm used in Guerrero et al. (2015) to accommodate the age structure of the cell populations we deal with here (Guerrero and Alarcón, 2015). For simplicity, we restrict our description of the algorithm to a homogeneous cell population. Its generalisation to heterogeneous populations composed of a variety of cellular types is straightforward.

Similarly to the procedure described in Guerrero and Alarcón (2015), we start by defining the population vector $N(t)=\{n_{a_{j}}(t),j\in J(t)\}$, where J(t) is the set of indexes which label all those age groups which, at time, *t*, are represented within the population, i.e. all those age groups such that $n(a=a_{j},t)>0$. Having defined N(t), we summarise the numerical algorithm:

1. Set initial conditions: $c(t=0)=c_{0}$, $n(a,t=0)=f(a)\Rightarrow N(t)=\{f_{a_{j}},j\in J(t=0)\}$, $N(t=0)=\int_{0}^{\infty }f(a)\mathrm{da}$. We also set the value of the ratio of the SCF-regulating enzymes $p_{6}/p_{3}$.
2. At some later time, *t*, the system is characterised by the quantities *c*(*t*), N(t), J(t), and *N*(*t*)
3. Generate two random numbers, *z*_1_ and *z*_2_, uniformly distributed in the interval (0,1)
4. Use *z*_1_ to calculate the exponentially distributed waiting time to the next event: $\tau =\frac{1}{a_{0}(t)}\;\log\;\frac{1}{z_{1}}$ where $a_{0}(t)=\sum_{j\in J(t)}(W_{1}(n_{a_{j}})+W_{2}(n_{a_{j}}))$. The rates *W*_1_ and *W*_2_ are given in Table 2
5. Update the oxygen concentration at c(t+τ) by solving the associated ODE (1) between (t,t+τ) taking *c*(*t*) as initial condition. We use a 4 stage Runge–Kutta solver
6. Update the age-dependent birth rate for each j∈J, i.e. $b(a)=\tau_{p}^{-1}H(a-a_{G1/s}(c(t+\tau ),p_{6}/p_{3}))$. The quantity $a_{G1/s}(c(t+\tau ),p_{6}/p_{3})$ is given by Eq. (13)
7. Use *z*_2_ to determine which event occurs (i.e. birth or death within the *j*th sub-population) at time t+τ: event *l* occurs with probability
  $$\overset{l}{\underset{k=1}{\sum }}W_{k}\leq z_{2}a_{0}(t)<\overset{J}{\underset{k=l+1}{\sum }}W_{k},$$
  where J=card(J(t)) and $W_{2(j-1)+1}=W_{1}(n_{a_{j}})$ and $W_{2(j-1)+2}=W_{2}(n_{a_{j}})$
8. If the randomly chosen event is l=2(j−1)+1 (i.e. cell proliferation within age group *j*), then $n_{a_{j}+\tau }(t+\tau )=n_{a_{j}}(t)-1$, $n_{a_{k}+\tau }(t+\tau )=n_{a_{k}}(t)$ for all k≠j, and $n_{a=0}(t+\tau )=n_{a=0}(t)+2$
9. If the randomly chosen event is l=2(j−1)+2 (i.e. cell death within age group *j*), then $n_{a_{j}+\tau }(t+\tau )=n_{a_{j}}(t)-1$, $n_{a_{k}+\tau }(t+\tau )=n_{a_{k}}(t)$ for all k≠j
10. Finally, we update the set J(t+τ), i.e. the set of age groups for which $n_{a_{j}+\tau }(t+\tau )>0$
11. Steps 3 to 10 are repeated until some stopping condition (e.g. t≥T) is satisfied

Note that Step 5 does not involve the use a stochastic method of integration of the ODE which rules the time evolution of the oxygen concentration, Eq. (1). This is due to the fact that, within the time interval (t,t+τ], the population stays constant, so that Eq. (1) can be solved by means of a non-stochastic solver.

### 4.2 Steady-state of a homogeneous population: mean-field analysis

Before proceeding further, in order to check the numerical algorithm proposed in Section 4.1, we analyse how it compares with results regarding the steady-state of the mean-field limit of a homogeneous (i.e. composed by one cellular type only) population Hoppensteadt (1975). The mean-field equations associated with the stochastic multi-scale model are:

(23) $$\frac{\mathrm{dc}}{\mathrm{dt}}=S-\mathrm{kNc},\frac{\partial Q}{\partial t}+\frac{\partial Q}{\partial a}=-(\nu +b(a))Q$$

with boundary condition:

Q(a=0,t)=2∫b(a)Q(a,t)da.

and birth rate given by:

$$b(a)=\tau_{p}^{-1}H(a-a_{G1/S}(c,p_{6}/p_{3}))$$

with $a_{G1/S}(c,p_{6}/p_{3})$ is given by Eq. (13)

According to Hoppensteadt (1975), in order to ascertain whether a steady-state solution, i.e. whether the system settles onto an age distribution where the proportion of cells of each age does not change, we seek for a separable solution: Q(a,t)=A(a)T(t). Defining μ(a)=ν+b(a), we obtain:

(24) $$\frac{1}{T}\frac{\mathrm{dT}}{\mathrm{dt}}=-\frac{1}{A}(\frac{\mathrm{dA}}{\mathrm{da}}+A\mu (a))=\sigma$$

with σ= cnt. to be determined. Q(a,t) is therefore given by:

(25) $$Q(a,t)=A(a=0)\exp(\sigma (t-a)-\int_{0}^{a}\mu (y)\mathrm{dy})$$

The value of the parameter *σ* is obtained by means of the characteristic equation obtained by introducing Eq. (25) with *a*=0 into the boundary condition:

(26) $$1=2\int_{0}^{\infty }b(a)\exp(-\sigma a-\int_{0}^{a}\mu (y)\mathrm{dy})\mathrm{da}$$

After some algebra, the characteristic equation (26) reads:

(27) $$2\frac{\tau_{p}^{-1}e^{-(\sigma +\nu )a_{G1/S}}}{\sigma +\nu +\tau_{p}^{-1}}=1$$

From Eq. (27) we obtain the condition for the system to be in equilibrium, i.e. σ=0. Substituting σ=0 in Eq. (26):

(28) $$R_{0}\equiv 2\frac{\tau_{p}^{-1}e^{-\nu a_{G1/S}}}{\nu +\tau_{p}^{-1}}=1$$

where *R*_0_ is the average number of offspring per cell at equilibrium: if $R_{0}>1$ the system grows exponentially, $R_{0}<1$ the system dwindles, and if $R_{0}=1$ the population remains constant. The equilibrium condition $R_{0}=1$ allows us to find the value of $a_{G1/S}$ for which such equilibrium exists:

(29) $$a_{G1/S}(c_{\infty },p_{6}/p_{3})=-\frac{1}{\nu }\log(\frac{\tau_{p}(\nu +\tau_{p}^{-1})}{2})$$

For this quantity to be positive $\tau_{p}\nu <1$ must hold. This condition states that for a steady state to be reached, the average waiting time to division after the G1/S transition, *τ*_*p*_, must be smaller than the average life span of the cell, *ν*^−1^. Eq. (29) determines the stationary value of the oxygen concentration, $c_{\infty }$.

The long-time dynamics of Eq. (23) can therefore be summarised as follows: given the values of *τ*_*p*_, *ν*, and the cell-cycle parameters (see Table B2 in Appendix B), the population evolves and consumes oxygen until the oxygen concentration reaches a steady value $c_{\infty }$. At this point, the population of resident cells has settled onto a steady state where its age structure does not change. The total number of cells is also constant and given by (see Eq. (23)):

(30) $$N_{\infty }=\frac{S}{{\mathrm{kc}}_{\infty }}$$

**Fig. 9:**
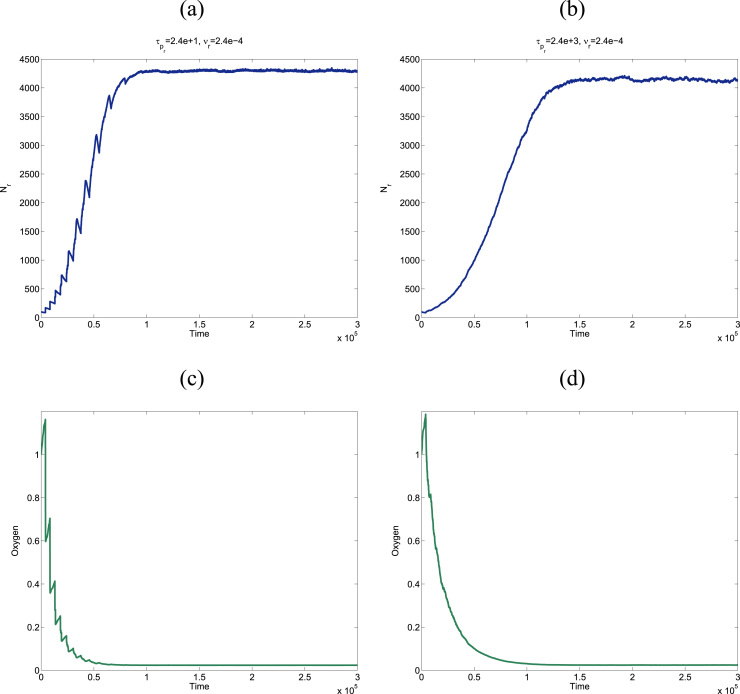
Plots showing simulation results of the stochastic multi-scale dynamics of a cell population. These plots show how, in agreement with our steady-state analysis, the population evolves until it reaches a steady-state where the population of resident cells fluctuates around its associated carrying capacity Eq. (30). Colour code: blue lines show the total resident cell population at time *t*, *N*(*t*) (panels (a) and (b)). Green lines (panels (c) and (d)) show the associated oxygen concentration, *c*(*t*). The results shown in this figure correspond to a single realisation of the process. Parameter values: $\nu =2.4\cdot 10^{-4}$, $S=1.57\cdot 10^{-2}$, $k=1.57\cdot 10^{-4}$, $\tau_{p}=2.4\cdot 10^{1}$ in panels (a) and (c), and $\tau_{p}=2.4\cdot 10^{3}$ in panels (b) and (d). (For interpretation of the references to color in this figure caption, the reader is referred to the web version of this paper.)

In order to verify the age-structured SSA proposed in Section 4.1, we compare its results with the mean-field predictions, which should be in agreement with the stochastic behaviour of the system for large values of the carrying capacity, $N_{\infty }$. Results are shown in Fig. 9. We observe that, as predicted by our steady-state analysis, the stochastic simulations show how the resident population goes through an initial (oxygen-rich) phase of exponential growth. As the population grows, oxygen is depleted and the resident population eventually saturates onto a number of cells which fluctuates around the mean-field prediction of the carrying capacity (Eq. (30)).

**Fig. 10:**
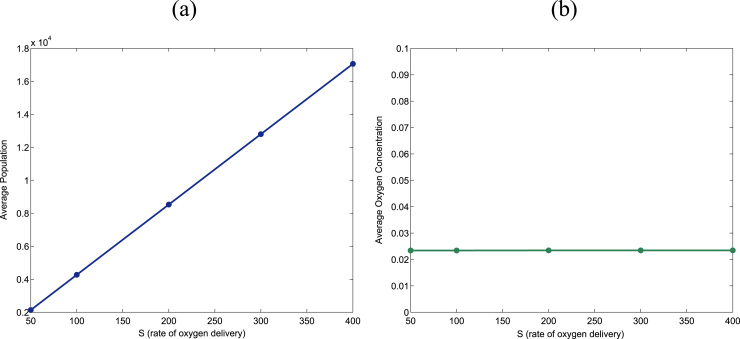
Plots showing simulation results of the stochastic multi-scale dynamics of a cell population. These plots show how, in agreement with our steady-state analysis, the average population at equilibrium increases linearly as the rate of oxygen delivery, *S*, is changed (plot (a)). Also, in accordance with our mean-field theory, the average, steady-state oxygen concentration remains unchanged as *S* increases. Parameter values: $\nu =2.4\cdot 10^{-4}$, $k=1.57\cdot 10^{-4}$, $\tau_{p}=2.4\cdot 10^{1}$ in panels (a) and (c), and $\tau_{p}=2.4\cdot 10^{3}$ in panels (b) and (d). Average has been performed over 100 realisations.

Further verification of the validity of our numerical methodology is provided in Fig. 10. Our mean-field theory predicts that, everything else remaining unchanged, the average steady-state population should increase linearly with the rate of oxygen delivery, *S* (see Eq. 30). By contrast, the average equilibrium oxygen concentration does not depend on *S*, as shown in Eq. (29), and, consequently, it should stay constant upon increasing the rate of oxygen delivery. Fig. 10 shows that our simulations agree with these mean-field predictions.

## 5 Quasi-neutral competition within heterogeneous populations

A problem of fundamental importance in several biological and biomedical contexts is that of a population composed by a heterogeneous mixture of coexisting cellular types. A particularly relevant example of such a situation is that of cancer, where heterogeneity within the cancer cell population is assumed to be a major factor in the evolutionary dynamics of cancer as well as the emergence of drug resistance (Gatenby and Vincent, 2003, Merlo et al., 2006, Gillies et al., 2012, Greaves and Maley, 2012). Within this context, we are interested in (i) exploring the stability of a co-existing heterogeneous population and (ii) the effects such heterogeneity has on the long-term effects a cell-cycle dependent therapy.

The origins of heterogeneity of cancer cell populations is normally attributed to genetic variability arising from chromosomal instability and increased mutation rate (Merlo et al., 2006). Our discussion of the model of the G1/S transition (see Section 3) suggests a different source of heterogeneity associated with stochastic effects due to intrinsic fluctuations. We have shown, by means of both the SCQSSA analysis and stochastic simulations, that the relative abundance of SCF-regulating enzymes, which is quantified in our analysis by the ratio $p_{6}/p_{3}$ (see Sections 3.5.1, 3.6 and Appendix A). In Section 3.5.1 we have shown that the timing of the G1/S transition, and, consequently, the overall birth rate is strongly affected by changes in this quantity. In view, of this we associate heterogeneity to a distribution of the abundance of such enzymes, whereby we associate cell phenotypes with different values of the ratio $p_{6}/p_{3}$. We further assume that this heterogeneity is hereditary, i.e. daughter cells inherit the value of the ratio $p_{6}/p_{3}$ from their mother.

### 5.1 Competition between two sub-populations

To proceed further, we consider the case of the birth-and-death dynamics of a heterogeneous population composed by two sub-populations, $n_{1}(a,t)$ and $n_{2}(a,t)$, competing by a common resource, *c*(*t*), which regulates the rate of progression of the cell-cycle of each cell type. The stochastic dynamics of the whole population is determined by the associated multi-scale master equation:

(31) $$\frac{\partial P(n_{1},n_{2},a,t)}{\partial t}+\frac{\partial P(n_{1},n_{2},a,t)}{\partial a}=W_{1}(n_{1}+1,n_{2},a,t)\;\times P(n_{1}+1,n_{2},a,t)\;+W_{2}(n_{1},n_{2}+1,a,t)\;\times P(n_{1},n_{2}+1,a,t)\;-(W_{1}(n_{1},n_{2},a,t)\;+W_{2}(n_{1},n_{2},a,t))\;\times P(n_{1},n_{2},a,t).$$

where $W_{1}(n_{1},n_{2},a,t)=(\nu_{1}+b_{1}(a))n_{1}(a,t)$ and $W_{2}(n_{1},n_{2},a,t)=(\nu_{2}+b_{2}(a))n_{1}(a,t)$, *ν*_1_ and $b_{1}(a)$ are the (age-independent) death rate and the birth rate of the resident population, and *ν*_2_ and $b_{2}(a)$ are the (age-independent) death rate and the birth rate of the invader. The quantities $b_{1}(a)$ and $b_{2}(a)$ are determined by the (oxygen-dependent) rate of cell-cycle progression of each sub-population:

(32) $$b_{1}(a)=\tau_{p_{1}}^{-1}H(a-a_{G1/S}(c,p_{6_{1}}/p_{3_{1}})),$$

(33) $$b_{2}(a)=\tau_{p_{2}}^{-1}H(a-a_{G1/S}(c,p_{6_{2}}/p_{3_{2}}))$$

$a_{G1/S}(c,p_{6}/p_{3})$ is given by Eq. (13). The concentration of resource (oxygen), *c*(*t*), is determined by the following ODE:

(34) $$\frac{\mathrm{dc}}{\mathrm{dt}}=S-(k_{1}N_{1}+k_{2}N_{2})c$$

where $N_{i}(t)=\int_{0}^{\infty }n_{i}(a,t)\mathrm{da}$ for *i*=1,2. The associated initial conditions are given by:

$$P(n_{1},n_{2},a,t=0)=\delta (n_{1}(a,t=0)-n_{0_{1}}(a))\delta (n_{2}(a,t=0)-n_{0_{2}}(a))$$

In the remaining part of this section we will consider two cellular populations, resident and invader. Each of these phenotypes are determined in terms of the values four parameters. The resident cells are characterised by two population-dynamics parameters, namely, the average time to division after the G1/S transition, $\tau_{p_{1}}$, and the death rate, *ν*_1_. We consider two further parameters, $p_{3_{1}}$ and $p_{6_{1}}$, associated with the cell-cycle progression dynamics of the resident cells (see Section 3.4). Similarly, the invader is characterised by the corresponding parameters: $\tau_{p_{2}}$, *ν*_2_, $p_{3_{2}}$ and $p_{6_{2}}$.

### 5.2 Mean-field coexistence versus quasi-neutral stochastic competition

We proceed to analyse the conditions under which two populations are capable of long-term coexistence. In particular, we analyse a scenario in which the mean-field description predicts long -term coexistence between within a heterogeneous population leads to mutual exclusion of all strands but one through so-called quasi-neutral stochastic competition (Lin et al., 2012, Kogan et al., 2014, Guerrero et al., 2015).

The starting point for our study is the mean-field analysis carried out in Section 4.2. According to these results, (mean-field) populations evolve until a concentration of oxygen, $c_{\infty }$, is reached so that the associated replication number $R_{0_{i}}(c_{\infty })=1$, *i*=1,2. The replication number of either population is given by:

$$R_{0_{1}}=2\frac{\tau_{p_{1}}^{-1}e^{-\nu_{1}a_{G1/S_{1}}(c_{\infty },p_{6_{1}}/p_{3_{1}})}}{\nu_{1}+\tau_{p_{1}}^{-1}}\;\mathrm{and}\;R_{0_{2}}=2\frac{\tau_{p_{2}}^{-1}e^{-\nu_{2}a_{G1/S_{2}}(c_{\infty },p_{6_{2}}/p_{3_{2}})}}{\nu_{2}+\tau_{p_{2}}^{-1}}$$

Therefore, our theory predicts that, provided that there exists $c_{\infty }$ such that:

(35) $$a_{G1/S_{1}}(c_{\infty },p_{6_{1}}/p_{3_{1}})=-\frac{1}{\nu_{1}}\log(\frac{\tau_{p_{1}}(\nu_{1}+\tau_{p_{1}}^{-1})}{2}),a_{G1/S_{2}}(c_{\infty },p_{6_{2}}/p_{3_{2}})=-\frac{1}{\nu_{2}}\log(\frac{\tau_{p_{2}}(\nu_{2}+\tau_{p_{2}}^{-1})}{2}),$$

is satisfied, long-term coexistence ensues, since the whole system (oxygen, resident and invader) is able to evolve to a state where both populations are in equilibrium (in the sense that $R_{0_{i}}(c_{\infty })=1$ for both populations) with the same concentration of oxygen. Furthermore, the number of resident cells, *N*_1_, and the number of invaders, *N*_2_, satisfy:

(36) $$k_{1}N_{1}+k_{2}N_{2}=\frac{S}{c_{\infty }}$$

Thus, the mean-field theory predicts that there exist a continuous of fixed points. The eventual convergence on to a particular point along the line of fixed points Eq. (36) depends on the initial conditions. Eq. (36) can be further simplified by assuming $k_{1}=k_{2}=k$, in which case $N_{1}+N_{2}=K$, where $K\equiv S/({\mathrm{kc}}_{\infty })$ is the carrying capacity.

This mean-field scenario is the basis for the study of the long-term stochastic dynamics of two populations which satisfy Eq. (35), which are equivalent to $R_{0_{1}}=R_{0_{2}}=1$. The reproduction number is the average number of offspring per cell. We know from elementary considerations (Grimmett and Stirzaker, 2001) that the value of such quantity allows us to classify birth-death/branching processes. If $R_{0}>1$ the population grows, on average, exponentially and has a finite probability of eventual survival. In this case, the process is referred to as super-critical. If $R_{0}<1$ the population undergoes exponential decline (on average) and the extinction probability is equal to 1. The last case, in which $R_{0}=1$, the so-called critical case, the population, on average, stays constant. However, due to effects of noise, extinction occurs with probability 1, with the probability of survival up to time *t* asymptotically tends to $P_{S}(t)∼t^{-1}$ (Kimmel and Axelrod, 2002). In our case, both the resident and the invader undergo a critical stochastic dynamics, where, once the steady state has established itself, the population evolves very close to the mean-field line of fixed points $N_{1}+N_{2}=K$ until fixation of one of the species (and, consequently, extinction of the other) occurs.

**Fig. 11:**
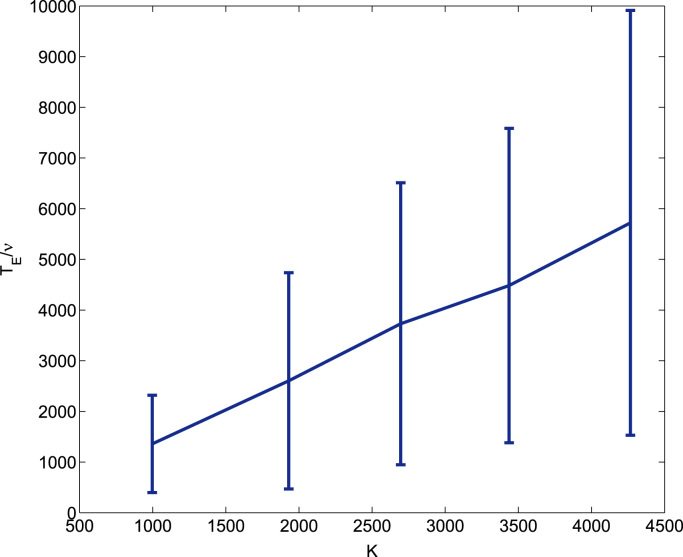
Simulation results corresponding to the competition between the sub-populations of a heterogeneous populations. We show how the average extinction time of either population, *T*_*E*_, varies as the carrying capacity, *K*, changes. We observe that the dependence is linear. For simplicity, the two sub-populations are assumed to be identical (i.e. with the same characteristic parameter values for both the intracellular dynamics (cell-cycle) and the population-level dynamics (birth and death rates)). The carrying capacity $K=\frac{S}{k_{1}c_{\infty }}$ by varying the death rates of both populations. The values of the death rates are $\nu =1.0\cdot 10^{-4},\;0.83\cdot 10^{-4},\;0.73\cdot 10^{-4},\;0.625\cdot 10^{-4},\;0.417\cdot 10^{-4}$, which correspond to $K=0.9969\cdot 10^{3},1.9301\cdot 10^{3},2.6956\cdot 10^{3},3.4367\cdot 10^{3},4.2661\cdot 10^{3}$, respectively. Averages are done over 500 realisations of the hybrid stochastic model.

In order to numerically check this scenario, we could proceed to estimate the survival probability at time *t*, *P*_*S*_(*t*). However, since this quantity exhibits a fat-tail behaviour, this would be computationally costly. A more efficient method is to resort to the asymptotic of the extinction time with system size, which in this case can be identified with the carrying capacity, *K* (Hidalgo et al., 2015). Typically, a quasi-neutral competition is associated with an algebraic dependence of the average extinction time of either population, *T*_*E*_, on the system size, in this case determined by the carrying capacity, *K* Doering et al., 2005, Kogan et al., 2014, Lin et al., 2012. In Fig. 11 we plot simulation results for the competition between two identical populations. In particular, we study how the average extinction time of either population, *T*_*E*_, varies as the carrying capacity is changed. We observe that this quantity exhibits a linear dependence on the carrying capacity:

(37) $$T_{E}∼K,$$

Our scenario produces the same qualitative results as Lin et al. (2012) and Kogan et al. (2014) who studied the average extinction time of birth-and-death processes engaged in quasi-neutral competition. In both papers, the average extinction time was reported to depend linearly on system size.

## 6 Study of the effects of cell-cycle-dependent therapy

In Section 5 we have analysed how the dependence of the cell-cycle progression on the concentration of SCF-activating and SCF-inactivating enzymes allows us to engineer heterogeneous populations where invasion and coexistence may occur. In this section, we further explore the ability of inducing quiescence by varying the ratio between SCF-activating and SCF-inactivating enzymes, this time in connection with the ability of such populations to withstand the effects of cell-cycle-dependent therapy (Powathil et al., 2012, Gabriel et al., 2012, Billy and Clairambault, 2013, Powathil et al., 2013).

In particular, we consider a scenario where two cellular populations coexist. Initially, one of these populations consists of a set of cells actively progressing through the cell-cycle which have reached a steady state characterised by the mean-field equilibrium Eqs. (28), (29), (30). The second population consists of quiescent cells whose cell-cycle is locked into the G0 phase and therefore do not proliferate. These cells are further assumed to undergo apoptosis at a very slow rate. More specifically, the ratio SCF-activating and SCF-inactivating enzymes in the quiescent cell population is such that, for the steady-state level of oxygen for the active cells (see Eq. (29)), cells are locked into the G1-fixed point (see Fig. 6).

In this section we show that the presence of a quiescent population within a heterogeneous population may lead to resistance to cell-cycle-dependent therapy. In particular, we show that, whereas such therapies effectively reduce or even eradicate the active cell population, the feedback between the therapy-induced decrease in cell numbers and the associated increase in oxygen availability can yield to the quiescent population to enter the active state and thus regrow the population. In this sense, we claim that the quiescent population has a *stem-cell-like* effect whereby, under the action of therapeutic agent, can repopulate the system Alarcón and Jensen, 2010.

### 6.1 Mean-field analysis

We start our mean field analysis by considering a heterogeneous population composed by cells of two types: type 1 and type 2 cells. Type 1 consists of cells with values of $p_{3}\equiv p_{3_{1}}$ and $p_{6}\equiv p_{6_{1}}$ (see Section 3.4) such that cells are actively progressing through the cell-cycle. Type 2 cells are characterised by values of $p_{3}\equiv p_{3_{2}}$ and $p_{6}\equiv p_{6_{2}}$ so that they are locked in G0 (i.e. not cycling). The associated mean-field dynamics are given by:

(38) $$\frac{\mathrm{dc}}{\mathrm{dt}}=S-k_{1}(N_{1}+N_{2})c,$$

(39) $$\frac{\partial Q_{1}}{\partial t}+\frac{\partial Q_{1}}{\partial a}=-(\nu_{1}+b_{1}(a))Q_{1}$$

(40) $$\frac{\partial Q_{2}}{\partial t}+\frac{\partial Q_{2}}{\partial a}=-\nu_{2}Q_{2}$$

with boundary conditions:

$$Q_{1}\left(a=0,t\right)=2\int b_{1}(a)Q_{1}(a,t)\mathrm{da},\;Q_{2}(a=0,t)=0.$$

$N_{1}(t)$ and $N_{2}(t)$ are the total cell population of type 1 and type 2 cells, respectively:

$$N_{1}(t)=\int Q_{1}(a,t)\mathrm{da},\;N_{2}(t)=\int Q_{2}(a,t)\mathrm{da}.$$

For simplicity, we assume that both populations consume oxygen at the same rate, *k*_1_. We further assume that $\nu_{2}⪡\nu_{1}$, i.e. type 2 (quiescent) cells die at a much slower rate than type 1 (active) cells. In Section 4.2, we have already analysed under which conditions Eqs. (38), (39), (40) reach a steady state:

(41) $$R_{0_{1}}\equiv 2\frac{\tau_{p_{1}}^{-1}e^{-\nu_{1}a_{G1/S_{1}}}}{\nu_{1}+\tau_{p_{1}}^{-1}}=1$$

where $R_{0_{1}}$ is the average number of offspring per cell of type 1 at equilibrium. The associated equilibrium value of $a_{G1/S_{1}}$ is then given by:

(42) $$a_{G1/S_{1}}(c_{\infty },p_{6_{1}}/p_{3_{1}})=-\frac{1}{\nu_{1}}\log(\frac{\left(\tau_{p_{1}}\nu_{1}+1\right)}{2})$$

which determines the steady-state value of the oxygen concentration $c_{\infty }$. At this point, the population of resident cells has settled onto a steady state where its age structure does not change. The total number of cells is also approximately constant and given by:

(43) $$N_{1_{\infty }}+N_{2}≃\frac{S}{k_{1}c_{\infty }}$$

where we have used the fact that $\nu_{2}⪡\nu_{1}$. The cell-cycle parameters $p_{3_{2}}$ and $p_{6_{2}}$ have been chosen so that, for $c=c_{\infty }$, $a_{G1/S_{2}}(c_{\infty },p_{6_{2}}/p_{3_{2}})\to \infty$, i.e. type 2 cells are initially locked into G0 (see Fig. 6).

Once the population reaches this therapy-free quasi-equilibrium state, we assume that a therapy which only acts on proliferating cells is administered. Examples of such therapies abound in cancer treatment and can take the form of cell-cycle specific drugs or radiotherapy (Powathil et al., 2012, Gabriel et al., 2012, Billy and Clairambault, 2013, Powathil et al., 2013). We characterise the efficiency of the therapy by the so-called survival fraction, *F*_*S*_, i.e. the percentage of cells which survive the prescribed dose. For example, in radiotherapy *F*_*S*_ is usually taken to be given by the linear quadratic model: $\log\;F_{S}=-(\alpha D+\beta D^{2})$ where *D* stands for the radiation dosage expressed in Grays and *α* and *β* are cell type-specific parameters. In the present context, we do not specify any particular form of therapy and we simply take $F_{S}\in [0,1)$. Initially, the therapy only affects type 1 cells (since type 2 are not proliferating). Therapy affects the birth and death rates of the type 1 population, which now read:

(44) $$b_{T_{1}}(a)=\tau_{p_{1}}^{-1}F_{S}H(a-a_{G1/S_{1}}(c,p_{3_{1}},p_{6_{1}}))=F_{s}b_{1}(a)$$

(45) $$\nu_{T_{1}}(a)=\nu_{1}+(1-F_{s})b_{1}(a)$$

The resulting mean-field equation is given by:

(46) $$\frac{\partial Q_{1}}{\partial t}+\frac{\partial Q_{1}}{\partial a}=-(\nu_{1}+b_{1}(a))Q_{1}$$

(47) $$Q_{1}(a=0,t)=2F_{S}\int b_{1}(a)Q_{1}(a,t)\mathrm{da}$$

The action of therapeutic agent on the active population initially induces a decline of the population, which, in turn, involves an increase in the available oxygen concentration. The latter has the effect of accelerating the rate of progression of type 1 cells through the cell cycle. In the absence of the quiescent population, eventually both effects would find a balance and the population of active cells would settle onto a new equilibrium characterised by:

(48) $$R_{0_{T_{1}}}\equiv 2F_{S}\frac{\tau_{p_{1}}^{-1}e^{-\nu_{1}a_{G1/S_{T_{1}}}}}{\nu_{1}+\tau_{p_{1}}^{-1}}=1$$

or, equivalently:

(49) $$a_{G1/S_{1}}(c_{T_{\infty_{1}}},p_{3_{1}},p_{6_{1}})=-\frac{1}{\nu_{1}}(-\log F_{S}+\log(\frac{\left(\tau_{p_{1}}\nu_{1}+1\right)}{2}))$$

where $c_{T_{\infty_{1}}}$ is the equilibrium oxygen concentration for the type 2 population with therapy. Note that $a_{G1/S_{1}}(c_{T_{\infty_{1}}},p_{3_{1}},p_{6_{1}})<a_{G1/S_{1}}(c_{\infty },p_{6_{1}}/p_{3_{1}})$ and therefore $c_{T_{\infty_{1}}}>c_{\infty }$ since $a_{G1/S}$ is a decreasing function of the oxygen concentration. Re-oxygenation during cell-cycle dependent therapy, in particular, radiotherapy, has been predicted by other models (Kempf et al., 2015).

Consider now the effect of this process on the type 2 cell population which is initially quiescent. We know that hypoxia-induced arrest of the cell cycle is reversible (Alarcón et al., 2004, Bedessem and Stephanou, 2014), i.e. upon increase of the concentration of oxygen quiescent cells may re-enter the cell cycle and become proliferating. Re-entry of quiescent cells into the cell cycle is predicated upon a sufficient increase in the oxygen contraction: $c>c_{H_{2}}(p_{3_{2}},p_{6_{2}})$, where the critical oxygen concentration for type 2 cells, $c_{H_{2}}$, depends on the momenta $p_{3_{2}}$ and $p_{6_{2}}$ or, equivalently, on the concentration of SCF-activating and SCF-inactivating enzymes. Taking this property into account, one can devise a scenario in which $c_{T_{\infty_{1}}}>c_{H_{2}}(p_{3_{2}},p_{6_{2}})>c_{\infty }$, i.e the initial oxygen concentration is such that type 2 cells are quiescent, but, as the therapy is administered and proceeds to act upon the type 1 cells, the oxygen concentration increases until it reaches its critical re- entry concentration. At this point, type 2 cells abandon quiescence and become active and competition between type 1 and type 2 cells ensues.

In order to assess the long-time behaviour of the system, we first study the equilibrium of the type 2 cell population upon re-entry into cell-cycle progression. Its mean-field dynamics is given by:

(50) $$\frac{\partial Q_{2}}{\partial t}+\frac{\partial Q_{2}}{\partial a}=-(\nu_{2}+b_{2}(a))Q_{1}$$

(51) $$Q_{2}(a=0,t)=2F_{S}\int b_{2}(a)Q_{2}(a,t)\mathrm{da}$$

where $b_{2}(a)=\tau_{p_{2}}^{-1}H(a-a_{G1/S_{2}}(c,p_{3_{2}},p_{6_{2}}))$ and *c* is determined by Eq. (38). Recall that, upon re-entering cell-cycle progression, type 2 cells are no longer immune to the therapy. Although in general the survival fraction is type-dependent, for simplicity we assume that *F*_*S*_ has the same value for both cell types. The equilibrium condition is once again given in terms of the associated reproduction number, i.e. $R_{0_{T_{2}}}=1$ which yields:

(52) $$a_{G1/S_{2}}(c_{T_{\infty_{2}}},p_{3_{2}},p_{6_{2}})=-\frac{1}{\nu_{2}}(-\log F_{S}+\log(\frac{\left(\tau_{p_{2}}\nu_{2}+1\right)}{2}))$$

Since $\nu_{2}⪡\nu_{1}$, we have that $a_{G1/S_{2}}(c_{T_{\infty_{2}}},p_{3_{2}},p_{6_{2}})⪢a_{G1/S_{1}}(c_{T_{\infty_{1}}},p_{3_{1}},p_{6_{1}})$ (see Eqs. (49), (52)). The latter inequality implies that the equilibrium oxygen concentration for type 2 cell, $c_{T_{\infty_{2}}}$, is such that $c_{T_{\infty_{2}}}<c_{T_{\infty_{1}}}$. It is easy to argue that, in these conditions, the type 2 population out-competes the type 1 cells: for $c_{T_{\infty_{2}}}<c<c_{T_{\infty_{1}}}$, the growth rate of type 1 cells is positive whereas the growth rate of the type 2 cell population is negative (see Eq. (27)). This implies that, upon application of therapy and provided that $c_{T_{\infty_{1}}}>c_{H_{2}}(p_{3_{2}},p_{6_{2}})>c_{\infty }$ is satisfied, the type 1 population declines and it is replaced by the type 2 population.

### 6.2 Critical dosage

In order to gain some degree of control over the behaviour described in the previous section, it would be useful to provide an estimate of the critical dosage above which therapy-induced re-oxygenation is capable of activating quiescent cells. We characterise the therapy dose by means of the critical survival fraction, $F_{S_{C}}$. Recall that the characteristic equation for the oxygen-dependent growth rate, $\sigma_{1}(c)$, of the population of active cells is given by:

(53) $$2F_{S}\frac{\tau_{p_{1}}^{-1}e^{-(\sigma_{1}+\nu_{1})a_{G1/S_{1}}(c)}}{\sigma_{1}+\nu_{1}+\tau_{p_{1}}^{-1}}=1$$

In order to activate the quiescent population the oxygen concentration must raise above the critical value $c_{H_{2}}(p_{3_{2}},p_{6_{2}})$. For the oxygen concentration to grow above this threshold $\sigma_{1}(c_{H_{2}})<0$ so that the active cell population continues to decline thus allowing the oxygen concentration to keep on raising. Therefore the critical value $F_{S_{C}}$ is such that $\sigma_{1}(c_{H_{2}})=0$, i.e.

(54) $$F_{S_{C}}=\frac{\nu_{1}+\tau_{p_{1}}^{-1}}{2\tau_{p_{1}}^{-1}e^{-\nu_{1}a_{G1/S_{1}}(c_{H_{2}}(p_{3_{2}},p_{6_{2}})}}$$

If $F_{S}<F_{S_{C}}$, the decrease in the active cell population is enough to provide enough oxygen for the quiescent population to become active.

This analysis implies that in heterogeneous populations which include quiescent sub-populations, the effect of cell-cycle-dependent therapy does not eradicate the population. Rather the following two scenarios are possible. If $c_{H_{2}}(p_{3_{2}},p_{6_{2}})>c_{T_{\infty_{1}}}$, type 2 cells do not become activated and become eventually extinct, and the type 1 cells settle onto the steady state prescribed by Eq. (49). If, by contrast, $c_{T_{\infty_{1}}}>c_{H_{2}}(p_{3_{2}},p_{6_{2}})>c_{\infty }$ is satisfied, type 2 cells out-compete type 1 cells and the system, composed entirely of type 2 cells, settles onto the steady state prescribed by Eq. (52). In this sense, quiescent cells have *stem-cell-like behaviour*, in the sense that they can repopulate the system. In this second scenario, therapy is not completely without virtue since therapy drives the system to be taken over by a slower-cycling (less aggressive) phenotype.

### 6.3 Simulation results

**Fig. 12:**
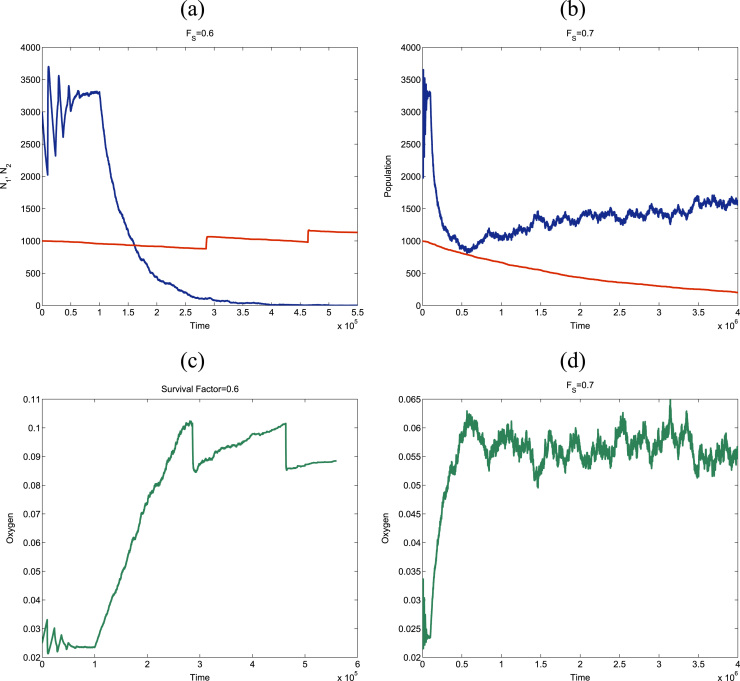
Stochastic simulation results showing typical realisations associated with rescue of quiescence cells (plots (a) and (c)) and recovery of the proliferating population (plots (b) and (d)) upon application of a cell-cycle dependent therapy. The efficiency of the therapy is characterised by the survival fraction, *F*_*S*_. Quiescence rescue is achieved when the survival fraction is set to a value which falls below the critical threshold, Eq. (54). If $F_{S}<F_{S_{C}}$ (plots (a) & (c)), the cell killing triggered by the therapy is enough to re-oxygenate the population above the activation threshold of the quiescent cells. By contrast, if $F_{S}>F_{S_{C}}$ (plots (b) and (d)), re-oxygenation is not enough to rescue latent cells from quiescence. Parameter values: $\nu_{1}=4.167\cdot 10^{-5}$, $\nu_{1}=4.167\cdot 10^{-7}$, $\tau_{p_{1}}=\tau_{p_{2}}=2.1\cdot 10^{-3}$, $\frac{p_{6_{1}}}{p_{3_{1}}}=1$, $\frac{p_{6_{1}}}{p_{3_{1}}}=0.989$. The subindex “1” corresponds to the active population whilst the subindex “2” denotes quantities associated with the quiescent population. The critical oxygen (as defined in 3.5.2, 3.6) is $c_{{\mathrm{cr}}_{1}}=0.023$ for the active cells and $c_{{\mathrm{cr}}_{2}}=0.1$ for the quiescent cells. Colour code: I all of the panels in this figure, blue (red) lines correspond to the time evolution of the total number of proliferating (quiescent) cells and green lines, to the time evolution of the oxygen concentration. (For interpretation of the references to color in this figure caption, the reader is referred to the web version of this paper.)

In order to check the accuracy of the mean-field analysis carried out in Section 6.2 regarding the critical survival fraction for rescue from quiescence. We start by showing (Fig. 12) two typical realisations of the stochastic population dynamics which illustrate the rescue mechanism. In these simulations, we first let the active population settle on to its steady state. We then apply a sustained therapy with constant survival fraction. A more aggressive treatment (*F*_*S*_=0.6 in Fig. 12) greatly affects the active population: the amount of active cells killed by the therapy induces re-oxygenation of the population above the critical oxygen level for activation of the quiescent population whereupon the quiescent cells become proliferating. In Figs. 12(a) and (c), we show that upon activation of the quiescent population, a competition between both populations ensues, which eventually leads to extinction of the active population. A less aggressive therapy (*F*_*S*_=0.7 in Fig. 12) also induces death of the active population and re-oxygenation. However, in this case, the latter is not intense enough to induce activation of the quiescent cells (see Fig. 12(d)) and therefore the active cells will repopulate the system as the quiescent population stays on its course to eventual extinction, as shown in Fig. 12(b).

**Fig. 13:**
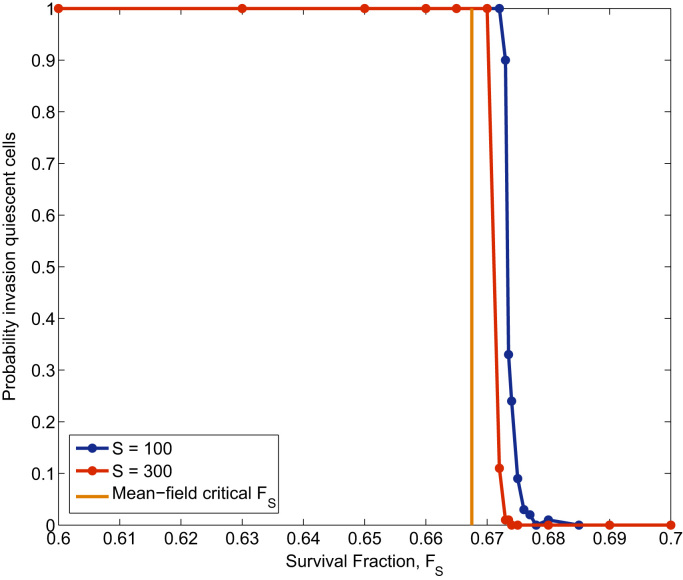
This figure shows stochastic simulation results regarding the variation of the probability of fixation of the quiescence population varies the efficiency of a cell-cycle dependent therapy changes. The efficiency of the therapy is measured in terms of the survival fraction *F*_*S*_. The orange vertical line represents the mean-field, theoretical critical survival fraction (Eq. 54). We observe that as the carrying capacity of the system increases, which in these simulations is achieved by increasing the rate of oxygen supply, *S*, the results of the stochastic simulations tend towards the mean-field prediction. Parameter values: $\nu_{1}=4.167\cdot 10^{-5}$, $\nu_{1}=4.167\cdot 10^{-7}$, $\tau_{p_{1}}=\tau_{p_{2}}=2.1\cdot 10^{-3}$, $\frac{p_{6_{1}}}{p_{3_{1}}}=1$, $\frac{p_{6_{1}}}{p_{3_{1}}}=0.989$. The subindex “1” corresponds to the active population whilst the subindex “2” denotes quantities associated with the quiescent population. The critical oxygen (as defined in 3.5.2, 3.6) is $c_{{\mathrm{cr}}_{1}}=0.023$ for the active cells and $c_{{\mathrm{cr}}_{2}}=0.1$ for the quiescent cells. Each data point corresponds to the average over 500 realisations of the stochastic population dynamics. (For interpretation of the references to color in this figure caption, the reader is referred to the web version of this paper.)

Fig. 13 shows simulation results for the variation of probability of fixation of the quiescent population as the survival fraction of the therapy, *F*_*S*_, changes. Our simulation results show qualitative agreement with our mean-field theory (see 6.1, 6.2): as the survival fraction increases (i.e. the therapy becomes less efficient), the probability of fixation abruptly decreases from almost certainty of fixation to almost certainty of extinction. We observe that our mean-field theoretical predicts a critical value for *F*_*S*_ slightly smaller than the observed when fluctuations due to finite size effects are present. However, we observe that, as the carrying capacity of the system is increased, the critical value of *F*_*S*_ converges to the mean-field value.

## 7 Discussion

In this paper, we have presented and studied a stochastic multi-scale model of a heterogeneous, resource-limited cell population. This model accounts for a stochastic intracellular dynamics (in this particular case, a model of the oxygen-regulated G1/S transition) and an age-structured birth-and-death process for the cell population dynamics. Both compartments are coupled by (i) a model for the time variation of resource (oxygen) abundance which regulates the rate of cell-cycle progression, and (ii) a model of the age-dependent birth rate which carries out the coupling between the intracellular and the cellular compartments (see Fig. 1 for a schematic representation of the model and Section 2 for a summary of the model formulation).

Our analysis of the stochastic dynamics of the oxygen-regulated G1/S transition, which is a generalisation of the mean-field model presented in Bedessem and Stephanou (2014), has revealed a number of previously unreported properties related to the presence of fluctuations. In particular, the optimal path theory and the quasi-steady state approximation allow us to explore the effect of the SCF-regulating enzymes on the timing of the G1/S transition. The relative abundance of SCF-activating and inhibiting enzymes regulates the rate at which cells reach the G1/S transition: excess of SCF-activating enzyme can delay the transition and even rendering the cell quiescent regardless of oxygen concentration beyond the predictions of the mean-field model (see 3.5.1, 3.5.2). Furthermore, we have shown that the effects on timing of the G1/S transition of the relative abundance of SCF-activating enzyme give rise to a scaling form dependence of the age to the transition, $a_{G1/S}$, whereby this quantity is a function of $a_{G1/S}(c,p_{6}/p_{3})$ which takes the form of Eq. (13) (see Section 3.6). Taken together, these results imply that stochasticity in the intracellular dynamics naturally generates variability within the population: an otherwise homogeneous population presents a distribution of birth rates induced by variability in the relative abundance of SCF-activating enzyme within the population of cells.

This variation in the duration of the cell-cycle allows us to analyse the dynamics of a stochastic heterogeneous population under resource limitation conditions. To this end, we consider populations formed by sub-populations of cells characterised by differing relative abundance of SCF-activating and inhibiting enzymes. We further assume that this heterogeneity is heritable (i.e. daughter cells inherited the ratio of SCF-regulating enzymes from their mother). In this scenario, we have shown sub-populations within heterogeneous population engage in quasi-neutral competition: sub-populations of cells get extinct in an average time which is of the order of the carrying capacity of the system (see Section 5.2). In the context of modelling cancer cells populations, where heterogeneity is a main contributor to the complex dynamics of cancer (Anderson et al., 2006, Merlo et al., 2006, Anderson and Quaranta, 2008, Gillies et al., 2012, Greaves and Maley, 2012), this result is of relevance since it allows us to estimate the rate at which sub-populations or clones disappear from the tumour. The fact that this rate is proportional to the inverse of the carrying capacity reveals a highly dynamic scenario where clones are quickly decaying and being replaced within the tumour. This can have a profound impact on a variety of evolutionary phenomena such as emergence of drug resistant phenotypes. From the modelling perspective, we should note that quasi-neutral competition is a purely stochastic scenario: the mean-field limit predicts coexistence between the corresponding cell types.

We have further explored the issue of emergence of drug resistant cell types by analysing a case study in which a quiescent population can be rescued from latency by the application of a cell-cycle dependent therapy. Examples of such therapies are radiotherapy or cytotoxic drugs designed to target cells in specific stages of cell cycle progression (Powathil et al., 2012). In the particular example analysed in Section 6, we have shown that in mixed population composed by active (proliferating) and quiescent cells, if the drug is not efficient enough (characterised in terms of its associated survival fraction, *F*_*S*_), quiescent cells are rescued from latency and eventually reach fixation within the population, i.e. the activated quiescent cells out-compete the original active cells until the latter population becomes extinct (Alarcón and Jensen, 2010). It is noteworthy the fact that in this process of quiescence rescue the original cancer population is replaced by a much more resistant population as the activated quiescent cells are less sensitive to the therapy than the original active cells.

Our results show that the methods and models presented in this paper are of great potential importance for the analysis of the complex dynamics of heterogeneous populations under resource limitation, in particular for the study of emergence of drug resistance in heterogeneous cancer cell populations. Several important issues have been left out of the present work. A major contributor to heterogeneity within a cancer cell population is spatial heterogeneity (Anderson et al., 2006, Gillies et al., 2012) which is closely related to micro-environmental heterogeneity. A further issue which should be analysed in depth concerns the scaling properties of the age to the G1/S transition (see Section 3.6), in particular whether this is a general property of the cell-cycle dynamics or rather a specific attribute of the stochastic model presented here. A thorough analysis of these issues falls beyond the scope of the present paper and are left for future research.

## Figures and Tables

**Table B1:** Dimensionless variables and parameters corresponding.

Dimensionless variables	Dimensionless parameters
$a=k_{7}\mathrm{ESt}$	ϵ=E/S
$q_{1}=Q_{1}/S$	[e2f]^tot=[e2f]tot/S
$q_{2}=Q_{2}/S$	$\kappa_{1}=k_{1}/(k_{7}{\mathrm{ES}}^{2})$
$q_{3}=Q_{3}/E$	$\kappa_{2}=k_{2}/(k_{7}{\mathrm{ES}}^{2})$
$q_{4}=Q_{4}/E$	$\kappa_{3}=k_{3}/(k_{7}\mathrm{ES})$
$q_{5}=Q_{5}/S$	$\kappa_{4}=k_{4}/(k_{7}S)$
$q_{6}=Q_{6}/E$	$\kappa_{5}=k_{5}/(k_{7}S^{2})$
$q_{7}=Q_{7}/E$	$\kappa_{6}=k_{6}/(k_{7}S^{2})$
$q_{8}=Q_{8}/S$	$\kappa_{8}=k_{8}/(k_{7}S)$
$q_{9}=Q_{9}/S$	$\kappa_{9}=k_{9}/(k_{7}S)$
$q_{10}=Q_{10}/S$	$\kappa_{10}=k_{10}/(k_{7}\mathrm{ES})$
	$\kappa_{11}=k_{11}/(k_{7}\mathrm{ES})$
	$\kappa_{12}=k_{12}/(k_{7}E)$
	$\kappa_{13}=k_{13}/(k_{7}{\mathrm{ES}}^{2})$
	$\kappa_{14}=k_{14}/(k_{7}\mathrm{ES})$
	$\kappa_{15}=k_{15}/(k_{7}E)$
	$\kappa_{16}=k_{16}/(k_{7}{\mathrm{ES}}^{2})$
	$\kappa_{17}=k_{17}/(k_{7}\mathrm{ES})$

**Table B2:** Equivalence between the parameters of the stochastic model of the G1/S transition (see Table 1) and the parameters of the associated mean-field model as formulated by Bedessem and Stephanou (2014).

Parameters	Parameters
$k_{1}=a_{1}*S$	$k_{11}=b_{2}$
$k_{2}=a_{3}*S*[H]$	$k_{12}=b_{3}/S$
$k_{3}=a_{2}$	$k_{13}=d_{2}*S$
$k_{5}=J_{2}*k_{4}S-k_{6}$	$k_{14}=d_{2}$
$k_{6}=e_{1}S/E$	$k_{15}=d_{1}/S$
$k_{8}=J_{1}*S-k_{9}$	$k_{16}=g_{1}{\left[E2F\right]}_{\mathrm{tot}}S$
$k_{9}=e_{2}/E$	$k_{17}=g_{1}$
$k_{10}=b_{1}$	

**Table B3:** Parameters values of the mean-field model of the hypoxia regulated G1/S transition proposed by Bedessem and Stephanou (2014).

Parameter	Value	Reference
$a_{1}$	0.51	Bedessem and Stephanou (2014)
$a_{2}$	1	Bedessem and Stephanou (2014)
$a_{3}H_{0}$	0.0085	Bedessem and Stephanou (2014)
$b_{1}$	0.018	Bedessem and Stephanou (2014)
$b_{2}$	0.5	Bedessem and Stephanou (2014)
$b_{3}$	1	Bedessem and Stephanou (2014)
$d_{1}$	0.2	Bedessem and Stephanou (2014)
$d_{2}$	0.1	Bedessem and Stephanou (2014)
$e_{1}$	1	Bedessem and Stephanou (2014)
$e_{2}$	14	Bedessem and Stephanou (2014)
$m_{⋆}$	10	Tyson and Novak (2001)
$J_{3},\;J_{4}$	0.04	Tyson and Novak (2001)
$g_{1}$	0.016	Bedessem and Stephanou (2014)
${\left[E2F\right]}_{\mathrm{tot}}$	1	Bedessem and Stephanou (2014)
S	10	
E	1	
$r_{\mathrm{cr}}$	0.04	

**Table B4:** Initial conditions used in simulation system (4), (B.36), (B.37), (7), (8), (9), (10), (11), (12) Fig. 4.

Parameter	Initial condition	Reference
*CycD*	0.1	Bedessem and Stephanou (2014)
*SCF*	0.9	Bedessem and Stephanou (2014)
*Rb*	1.0	Bedessem and Stephanou (2014)
*E2F*	0.1	Bedessem and Stephanou (2014)
*m*	5.0	Bedessem and Stephanou (2014)

**Table 1:** Reaction probability per unit time, $W_{i}\equiv W(X,r_{i},t),i=1,\ldots ,14$. The mass is assumed to grow following a logistic law: $m(a)=\frac{m_{*}{\mathrm{Ke}}^{\eta a}}{{\mathrm{Ke}}^{\eta a}-1}=\frac{m_{*}}{1-{\mathrm{Ke}}^{-\eta a}}$, where $K=1-\frac{m_{*}}{m(0)}$ and *a* is the age of the cell (i.e. the time elapsed since birth). According to Bedessem and Stephanou (2014), the level of active HIF-1 α , [H], depends exponentially on the extracellular oxygen concentration, c : $[H]=H_{0}e^{\beta_{1}(1-c)}$ . Furthermore, Following Novak and Tyson (2004), we assume that at each time, the active E2F, $\left[E2F_{A}\right]$, is the fraction of unphosphorylated free E2F factor, $[E2F]:[E2F_{A}]=\frac{\left({\left[E2F\right]}_{\mathrm{tot}}-[E2F_{\mathrm{Rb}}])[E2F\right]}{{\left[E2F\right]}_{\mathrm{tot}}}$ . The equilibrium between E2F–Rb complexes; free E2F and free Rb is given by Novak and Tyson (2004): $[E2F_{\mathrm{Rb}}]=[\mathrm{Rb}]=\frac{2{\left[E2F\right]}_{\mathrm{tot}}[\mathrm{Rb}]}{{\left[E2F\right]}_{\mathrm{tot}}+[\mathrm{Rb}]+\sqrt{{\left({\left[E2F\right]}_{\mathrm{tot}}+[\mathrm{Rb}]\right)}^{2}-4{\left[E2F\right]}_{\mathrm{tot}}[\mathrm{Rb}]}}$.

Variable	Description
*X*_1_, *X*_8_	Number of Cyclin D and Cyclin E molecules, respectively
*X*_2_, *X*_5_	Number of inactive and active SCF molecules, respectively
*X*_3_, *X*_6_	Number of SCF-activating and SCF-inactivating enzyme molecules, respectively
*X*_4_, *X*_7_	Number of enzyme-inactive SCF and enzyme-active SCF complexes, respectively
*X*_9_, *X*_10_	Number of free RbP and E2F molecules, respectively


Reaction probability p.u.t.	*r*_*i*_
$W_{1}=k_{1}-k_{2}[H]$	(1,0,0,0,0,0,0,0,0,0)
$W_{2}=k_{3}X_{1}$	(−1,0,0,0,0,0,0,0,0,0)
$W_{3}=k_{4}X_{2}X_{3}$	(0,−1,−1,1,0,0,0,0,0,0)
$W_{4}=k_{5}X_{4}$	(0,1,1,−1,0,0,0,0,0,0)
$W_{5}=k_{9}X_{8}X_{7}$	(0,1,0,0,0,1,−1,0,0,0)
$W_{6}=k_{6}X_{4}$	(0,0,1,−1,1,0,0,0,0,0)
$W_{7}=k_{7}X_{5}X_{8}X_{6}$	(0,0,0,0,−1,−1,1,0,0,0)
$W_{8}=k_{8}X_{8}X_{7}$	(0,0,0,0,1,1,−1,0,0,0)
$W_{9}=k_{10}m[E2F_{A}]=k_{10}m(1-\frac{X_{9}}{{\left[E2F\right]}_{\mathrm{tot}}})X_{10}$	(0,0,0,0,0,0,0,1,0,0)
$W_{10}=(k_{11}+k_{12}X_{5})X_{8}$	(0,0,0,0,0,0,0,−1,0,0)
$W_{11}=k_{13}$	(0,0,0,0,0,0,0,0,1,0)
$W_{12}=(k_{14}+k_{15}X_{1})X_{9}$	(0,0,0,0,0,0,0,0,−1,0)
$W_{13}=k_{16}$	(0,0,0,0,0,0,0,0,0,1)
$W_{14}=k_{17}X_{10}$	(0,0,0,0,0,0,0,0,0,−1)

**Table 2:** This table summarises the elementary events involved in the age-dependent birth-and-death process. Cells of age a produce offspring at a rate proportional to the age dependent birth rate, b(a), Eq. (18). We are assuming that upon cell division both cells are reset to a=0. Therefore, upon proliferation, one cell is removed from the population of age a and two cells are added to the population of age a=0. For simplicity, death is assumed to be age-independent and to occur at a rate proportional to the death rate, μ.

Event	Reaction	Transition rate, $W_{k}(n,a,t)$	$r_{k}$

Birth	n(a)→n(a)−1∅→n(a=0)=2	$W_{1}(n,a,t)=b(a)n(a)$	$r_{1}=-1$
Death	n(a)→n(a)−1	$W_{2}(n,a,t)=\mu n(a)$	$r_{2}=-1$

## Appendix A Summary of the semi-classical quasi-steady state approximation

In this appendix, we give a summary of the semi-classical quasi-steady state approximation (SCQSSA). For a more detailed presentation of the method, the reader is referred to Alarcón, 2014, de la Cruz et al., 2015.

Following Alarcón, 2014, we formulate the QSS approximation for the asymptotic solution of the CME obtained by means of large deviations/WKB approximations (Kubo et al., 1973, Alarcón and Page, 2007, Freidlin and Wentzell, 1998, Touchette, 2009). An alternative way to analyse the dynamics of continuous-time Markov processes on a discrete space of states is to derive an equation for the generating function, $G(p_{1},\ldots ,p_{n},t)$ of the corresponding probabilistic density:

(A.1) $$G(p_{1},\ldots ,p_{n},t)=\underset{x}{\sum }p_{1}^{X_{1}}p_{2}^{X_{2}}\cdots p_{n}^{X_{n}}P(X_{1},\ldots ,X_{n},t)$$

where $P(X_{1},\ldots ,X_{n},t)$ is the solution of the CME Eq. (3). $G(p_{1},\ldots ,p_{n},t)$ satisfies a partial differential equation (PDE) which can be derived from the CME. This PDE is the basic element of the so-called momentum representation of the Master Equation (Doi, 1976, Peliti, 1985, Assaf and Meerson, 2006, Assaf et al., 2010, Kang and Kurtz, 2013). Analytical solutions for the generating function PDE are seldom available and one needs to resort to approximate solutions, which are commonly obtained by means of the WKB asymptotic (Assaf et al., 2010). More specifically, the (linear) PDE that governs the evolution of the generating function can be written as:

(A.2) $$\frac{\partial G}{\partial t}=H_{k}\left(p_{1},\ldots ,p_{n},\partial_{p_{1}},\ldots ,\partial_{p_{n}}\right)G(p_{1},\ldots ,p_{n},t)$$

where the operator *H*_*k*_ is determined by the reaction rates of the CME (3). Furthermore, the solution to this equation must satisfy the normalisation condition $G(p_{1}=1,\ldots ,p_{n}=1,t)=1$ for all *t*. The operator *H* is obtained by multiplying both sides of the CME by $\prod_{i=1}^{n}p_{i}^{X_{i}}$ and summing up over all the possible values of $\left(X_{1},\ldots ,X_{n}\right)$.

Eq. (A.2) is a Schrödinger-like equation and, therefore, there is a plethora of methods at our disposal in order to analyse it. In particular, when the fluctuations are (assumed to be) small, it is common to use WKB methods (Kubo et al., 1973, Alarcón and Page, 2007, Gonze et al., 2002). This approach is based on the WKB-like Ansatz that $G(p_{1},\ldots ,p_{n},t)=e^{-S(p_{1},\ldots ,p_{n},t)}$. By substituting this Ansatz in Eq. (A.2) we obtain the following Hamilton–Jacobi equation for the function $S(p_{1},\ldots ,p_{n},t)$:

(A.3) $$\frac{\partial S}{\partial t}=-H_{k}(p_{1},\ldots ,p_{n},\frac{\partial S}{\partial p_{1}},\ldots ,\frac{\partial S}{\partial p_{n}})$$

Instead of directly tackling Eq. (A.3), we will use the so-called semi-classical approximation. We use the Feynman path-integral representation which yields a solution to Eq. (A.2) of the type (Peliti, 1985, Feynman and Hibbs, 2010, Kubo et al., 1973, Dickman and Vidigal, 2003, Elgart and Kamenev, 2004, Tauber et al., 2005):

(A.4) $$G(p_{1},\ldots ,p_{n},t)=\int_{0}^{t}e^{-S(p_{1},\ldots ,p_{n},Q_{1},\ldots ,Q_{n})}DQ(s)Dp(s),$$

where DQ(s)Dp(s) indicates integration over the space of all possible trajectories and $S(p_{1},\ldots ,p_{n},Q_{1},\ldots ,Q_{n})$ is given by (Kubo et al., 1973):

(A.5) $$S(p_{1},\ldots ,p_{n},Q_{1},\ldots ,Q_{n})=-\int_{0}^{t}(H_{k}(p_{1},\ldots ,p_{n},Q_{1},\ldots ,Q_{n})+\overset{n}{\underset{i=1}{\sum }}Q_{i}(s){\overset{˙}{p}}_{i}(s))\mathrm{ds}+\overset{n}{\underset{i=1}{\sum }}S_{0,i}(p_{i},Q_{i}),$$

The position operators in the momentum representation have been defined as $Q_{i}\equiv \partial_{p_{i}}$ with the commutation relation $[Q_{i},p_{j}]=S_{0,i}\delta_{i,j}$. $S_{0,i}(p_{i},Q_{i})$ corresponds to the action associated with the generating function of the probability distribution function of the initial value of each variable, $X_{i}(t=0)$, which are assumed to be independent random variables. The so-called semi-classical approximation consists of approximating the path integral in Eq. (A.4) by

(A.6) $$G(p_{1},\ldots ,p_{n},t)=e^{-S(p_{1},\ldots ,p_{n},t)}$$

where $p_{1}(t),\ldots ,p_{n}(t)$ are now the solutions of the Hamilton equations, i.e. the orbits which maximise the action *S*:

(A.7) $$\frac{{\mathrm{dp}}_{i}}{\mathrm{dt}}=-\frac{\partial H_{k}}{\partial Q_{i}}$$

(A.8) $$\frac{{\mathrm{dQ}}_{i}}{\mathrm{dt}}=\frac{\partial H_{k}}{\partial p_{i}}$$

where the pair (*Q*_*i*_,*p*_*i*_) are the generalised coordinates corresponding to chemical species i=1,…,n. These equations are (formally) solved with boundary conditions (Elgart and Kamenev, 2004)$Q_{i}(0)=x_{i}(0)$, $p_{i}(t)=p_{i}$, where $x_{i}(0)$ is the initial number of molecules of species *i*.

Eqs. (A.7), (A.8) are the starting point for the formulation of the semi-classical quasi-steady state approximation (SCQSSA) (Alarcón, 2014, de la Cruz et al., 2015). In order to proceed further, we assume, as per the Briggs–Haldane treatment of the Michealis–Menten model for enzyme kinetics (Briggs and Haldane, 1925, Keener and Sneyd, 1998), that the species involved in the system under scrutiny are divided into two groups according to their characteristic scales. More specifically, we have a subset of chemical species whose numbers, *X*_*i*_, scale as:

(A.9) $$X_{i}={\mathrm{Sx}}_{i},$$

where $x_{i}=O(1)$, whilst the remaining species are such that their numbers, *X*_*j*_, scale as:

(A.10) $$X_{j}={\mathrm{Ex}}_{j},$$

where $x_{j}=O(1)$. Key to our approach is the fact that *S* and *E* must be such that:

(A.11) $$\epsilon =\frac{E}{S}⪡1.$$

We further assume (de la Cruz et al., 2015) that the generalised coordinates, *Q*_*i*_, scale in the same fashion as the corresponding variable *X*_*i*_, i.e.

(A.12) $$Q_{i}={\mathrm{Sq}}_{i},$$

where $q_{i}=O(1)$. We refer to the variables belonging to this subset as *slow variables*. Similarly,

(A.13) $$Q_{j}={\mathrm{Eq}}_{j},$$

where $q_{j}=O(1)$, which are referred to as *fast variables*. Moreover, we assume that the moment coordinates, *p*_*i*_, are all independent of *S* and *E*, and therefore remain invariant under rescaling.

Under this scaling for the generalised coordinates, we define the following scale transformation for the Hamiltonian in Eq. (A.5):

(A.14) $$H_{k}(p_{1},\ldots ,p_{n},Q_{1},\ldots ,Q_{n})=k_{J}S^{k}E^{l}H_{\kappa }(p_{1},\ldots ,p_{n},q_{1},\ldots ,q_{n})$$

where *J* identifies the reaction with the largest order among all the reactions that compose the dynamics and *k*_*J*_ is the corresponding rate constant. In the case of the stochastic model of the G1/S transition *J*=7 (see Table 1), this reaction is order 3 whereas all the others are of lower order. The exponents *k* and *l* correspond to the number of slow and fast variables involved in the transition rate *W*_7_, respectively. In this particular case we have *k*=2 and *l*=1. The last step is to rescale the time variable so that a dimensionless variable, *a*, is defined such that:

(A.15) $$a=k_{J}S^{k-1}E^{l}t$$

It is now a trivial exercise to check that, upon rescaling, Eqs. (A.7), (A.8) read

(A.16) $$\frac{{\mathrm{dp}}_{i}}{\mathrm{da}}=-\frac{\partial H_{\kappa }}{\partial q_{i}},$$

(A.17) $$\frac{{\mathrm{dq}}_{i}}{\mathrm{da}}=\frac{\partial H_{\kappa }}{\partial p_{i}},$$

for the slow variables. By contrast, rescaling of the Hamilton equations corresponding to the subset of fast variables leads to:

(A.18) $$\epsilon \frac{{\mathrm{dp}}_{j}}{\mathrm{da}}=-\frac{\partial H_{\kappa }}{\partial q_{j}},$$

(A.19) $$\epsilon \frac{{\mathrm{dq}}_{j}}{\mathrm{da}}=\frac{\partial H_{\kappa }}{\partial p_{j}},$$

where *ϵ* is defined in Eq. (A.11). The QSS approximation consists on assuming that $\epsilon \frac{{\mathrm{dp}}_{j}}{\mathrm{da}}≃0$ and $\epsilon \frac{{\mathrm{dq}}_{j}}{\mathrm{da}}≃0$ in Eqs. (A.18), (A.19),

(A.20) $$-\frac{\partial H_{\kappa }}{\partial q_{j}}=0,$$

(A.21) $$\frac{\partial H_{\kappa }}{\partial p_{j}}=0,$$

resulting in a differential-algebraic system of equations which provides us with the semi-classical quasi-steady state approximation (SCQSSA).

## Appendix B Semi-classical quasi-steady state analysis of the stochastic model of the G1/S transition

In this appendix we derive the equations of the SCQSSA of the stochastic model of the G1/S transition as defined by the Master Equation (3) and the associated transition rates shown in Table 1. See Alarcón, 2014, de la Cruz et al., 2015 for details of the procedure. The associated generating function satisfies the following PDE:

(B.1) $$\frac{\partial G}{\partial t}=H(p_{1},\ldots ,p_{10},\partial_{p_{1}},\ldots ,\partial_{p_{10}})G(p_{1},\ldots ,p_{10},t)$$

where the operator $H(p_{1},\ldots ,p_{10},Q_{1},\ldots ,Q_{10})\equiv H(p,Q)$, where $Q_{i}\equiv \partial_{p_{i}}$ which satisfies the commutation relation $[Q_{i},p_{j}]=S_{0,i}\delta_{i,j}$, is given by:

(B.2) $$H(p,Q)=H_{1}(p,Q)+H_{2}(p,Q)+H_{3}(p,Q)+H_{4}(p,Q)+H_{5}(p,Q)+H_{6}(p,Q)$$

where the full Hamiltonian H(p,Q) has been separated into six parts, each corresponding to one of the reactions shown in Fig. 3. Thus,

(B.3) $$H_{1}(p,Q)=k_{4}(p_{4}-p_{2}p_{3})Q_{2}Q_{3}+k_{5}(p_{2}p_{3}-p_{4})Q_{4}+k_{6}(p_{3}p_{5}-p_{4})Q_{4},$$

and

(B.4) $$H_{2}(p,Q)=k_{9}p_{8}(p_{2}p_{6}-p_{7})Q_{7}Q_{8}+k_{7}p_{8}(p_{7}-p_{5}p_{6})Q_{5}Q_{6}Q_{8}+k_{8}p_{8}(p_{5}p_{6}-p_{7})Q_{7}Q_{8},$$

correspond to enzymatic activation and (CycE-mediated) inactivation of SCF (*X*_5_), respectively. $H_{3}(p,Q)$ through to $H_{6}(p,Q)$ are given by:

(B.5) $$H_{3}(p,Q)=(k_{1}-k_{2})(p_{1}-1)+k_{3}(1-p_{1})Q_{1},$$

(B.6) $$H_{4}(p,Q)=k_{10}m(p_{8}-1)p_{10}Q_{10}(1-\frac{p_{9}}{{\left[e2f\right]}_{\mathrm{tot}}}Q_{9})+(1-p_{8})Q_{8}(k_{11}+k_{12}p_{5}Q_{5}),$$

(B.7) $$H_{5}(p,Q)=k_{13}(p_{9}-1)+(1-p_{9})(k_{14}Q_{9}+k_{15}p_{1}Q_{1}Q_{9}),$$

(B.8) $$H_{6}(p,Q)=k_{16}(p_{10}-1)+k_{17}(1-p_{10})Q_{10},$$

which are the Hamiltonians associated with synthesis and degradation of CycD (*X*_1_), CycE (*X*_8_), Rb (*X*_9_), and E2F (*X*_10_), respectively.

By re-scaling the coordinate-like variables *Q*_*i*_ according to the scaling shown in Table B1 (see Section 3.4 and reference de la Cruz et al., 2015) and defining the dimensionless time as $a=k_{7}\mathrm{ESt}$, the associated Hamilton equations, Eqs. (A.7), (A.8)

(B.9) $$\frac{{\mathrm{dq}}_{1}}{\mathrm{da}}=\kappa_{1}-\kappa_{2}-\kappa_{3}q_{1}+\kappa_{15}(1-p_{9})q_{1}q_{9}$$

(B.10) $$\frac{{\mathrm{dq}}_{2}}{\mathrm{da}}=-\kappa_{4}p_{3}q_{2}q_{3}+\kappa_{5}p_{3}q_{4}+\kappa_{9}p_{8}p_{6}q_{7}q_{8}$$

(B.11) $$\epsilon \frac{{\mathrm{dq}}_{3}}{\mathrm{da}}=-\kappa_{4}p_{2}q_{2}q_{3}+(\kappa_{5}p_{2}+\kappa_{6}p_{5})q_{4}$$

(B.12) $$\epsilon \frac{{\mathrm{dq}}_{4}}{\mathrm{da}}=\kappa_{4}q_{2}q_{3}-(\kappa_{5}+\kappa_{6})q_{4}$$

(B.13) $$\frac{{\mathrm{dq}}_{5}}{\mathrm{da}}=\kappa_{6}p_{3}q_{4}-p_{8}p_{6}q_{5}q_{6}q_{8}+\kappa_{8}p_{8}p_{6}q_{7}q_{8}+\kappa_{12}q_{5}q_{8}(1-p_{8})$$

(B.14) $$\epsilon \frac{{\mathrm{dq}}_{6}}{\mathrm{da}}=\kappa_{9}p_{8}p_{2}q_{7}q_{8}-p_{8}p_{5}q_{5}q_{6}q_{8}+\kappa_{8}p_{8}p_{5}q_{7}q_{8}$$

(B.15) $$\epsilon \frac{{\mathrm{dq}}_{7}}{\mathrm{da}}=-\kappa_{9}p_{8}q_{7}q_{8}+p_{8}q_{5}q_{6}q_{8}-\kappa_{8}p_{8}q_{7}q_{8}$$

(B.16) $$\frac{{\mathrm{dq}}_{8}}{\mathrm{da}}=(\kappa_{9}(p_{2}p_{6}-p_{7})+\kappa_{8}(p_{5}p_{6}-p_{7}))q_{7}q_{8}+(p_{7}-p_{5}p_{6})q_{5}q_{6}q_{8}+$$

(B.17) +κ10mp10q10(1−p9q9[e2f]^tot)−κ11q8−κ12p5q5q8

(B.18) dq9da=κ10m(1−p8)p10q9q10[e2f]^tot+κ13−κ14q9−κ15p1q1q9

(B.19) dq10da=κ10m(p8−1)q10(1−p9q9[e2f]^tot)+κ16−κ17q10

(B.20) $$\frac{{\mathrm{dp}}_{1}}{\mathrm{da}}=-\kappa_{3}(1-p_{1})-\kappa_{15}(1-p_{9})p_{1}q_{9}$$

(B.21) $$\frac{{\mathrm{dp}}_{2}}{\mathrm{da}}=-\kappa_{4}(p_{4}-p_{2}p_{3})q_{3}$$

(B.22) $$\epsilon \frac{{\mathrm{dp}}_{3}}{\mathrm{da}}=-\kappa_{4}(p_{4}-p_{2}p_{3})q_{2}$$

(B.23) $$\epsilon \frac{{\mathrm{dp}}_{4}}{\mathrm{da}}=-\kappa_{5}(p_{2}p_{3}-p_{4})-\kappa_{6}(p_{3}p_{5}-p_{4})$$

(B.24) $$\frac{{\mathrm{dp}}_{5}}{\mathrm{da}}=-p_{8}(p_{7}-p_{5}p_{6})q_{6}q_{8}-\kappa_{12}(1-p_{8})p_{5}q_{8}$$

(B.25) $$\epsilon \frac{{\mathrm{dp}}_{6}}{\mathrm{da}}=-p_{8}(p_{7}-p_{5}p_{6})q_{5}q_{8}$$

(B.26) $$\epsilon \frac{{\mathrm{dp}}_{7}}{\mathrm{da}}=-(\kappa_{9}(p_{2}p_{6}-p_{7})+\kappa_{8}(p_{5}p_{6}-p_{7}))p_{8}q_{8}$$

(B.27) $$\frac{{\mathrm{dp}}_{8}}{\mathrm{da}}=-(\kappa_{9}(p_{2}p_{6}-p_{7})+\kappa_{8}(p_{5}p_{6}-p_{7}))p_{8}q_{7}-p_{8}(p_{7}-p_{5}p_{6})q_{5}q_{6}$$

(B.28) $$-(1-p_{8})(\kappa_{11}+\kappa_{12}p_{5}q_{5})$$

(B.29) dp9da=−κ10m(1−p8)p10p9q10[e2f]^tot−(1−p9)(κ14+κ15p1q1)

(B.30) dp10da=−κ10m(p8−1)p10(1−p9q9[e2f]^tot)−κ17(1−p10)

where ϵ=E/S⪡1 and the re-scaled parameters *κ*_*i*_ are given in Table B1. Applying the QSSA to the equations for the momenta *p*_3_, *p*_4_, *p*_6_ and *p*_7_ associated with the fast variables, we obtain:

(B.31) $$\epsilon \frac{{\mathrm{dp}}_{3}}{\mathrm{da}}=0\Rightarrow p_{4}=p_{2}p_{3},\;\epsilon \frac{{\mathrm{dp}}_{4}}{\mathrm{da}}=0\Rightarrow p_{4}=p_{5}p_{3}$$

(B.32) $$\epsilon \frac{{\mathrm{dp}}_{6}}{\mathrm{da}}=0\Rightarrow p_{7}=p_{5}p_{6},\;\epsilon \frac{{\mathrm{dp}}_{7}}{\mathrm{da}}=0\Rightarrow p_{7}=p_{2}p_{6}$$

which implies that $p_{2}=p_{5}$, which, in turn, yields that $p_{8}=1$ (see Eqs. (B.21), (B.24)). Furthermore, the QSSA applied to the equations for the generalised coordinates associated with the SCF-activating, *q*_3_, Eq. (B.11), and the enzyme-active SCF complex, *q*_7_, Eq. (B.15), yields:

(B.33) $$\epsilon \frac{{\mathrm{dq}}_{3}}{\mathrm{da}}=0\Rightarrow \kappa_{5}q_{4}-\kappa_{4}q_{2}q_{3}=-\kappa_{6}q_{4},\;\epsilon \frac{{\mathrm{dq}}_{6}}{\mathrm{da}}=0\Rightarrow \kappa_{8}q_{7}-q_{5}q_{6}=-\kappa_{9}q_{7},$$

which implies that $q_{2}+q_{5}=p_{c}$. Furthermore, since $q_{3}+q_{4}=p_{e_{1}}$ and $q_{6}+q_{7}=p_{e_{2}}$ are satisfied, we obtain that:

(B.34) $$q_{4}=\frac{p_{e_{1}}q_{2}}{q_{2}+\frac{\kappa_{5}+\kappa_{6}}{\kappa_{4}}},\;q_{7}=\frac{p_{e_{2}}q_{5}}{q_{5}+(\kappa_{8}+\kappa_{9})}$$

Finally, using Eqs. (B.31), (B.32), (B.33), (B.34), the SCQSSA equations for the stochastic model of the G1/S transition are:

(B.35) $$\frac{{\mathrm{dq}}_{1}}{\mathrm{da}}=\kappa_{1}-\kappa_{2}-\kappa_{3}q_{1}+\kappa_{15}(1-p_{9})q_{1}q_{9}$$

(B.36) $$\frac{{\mathrm{dq}}_{5}}{\mathrm{da}}=\frac{\kappa_{6}p_{3}p_{e_{1}}(p_{c}-q_{5})}{(p_{c}-q_{5})+\frac{\kappa_{5}+\kappa_{6}}{\kappa_{4}}}-\frac{\kappa_{9}p_{8}p_{6}p_{e_{2}}q_{5}q_{8}}{q_{5}+(\kappa_{8}+\kappa_{9})}$$

(B.37) dq8da=κ10mp10q10(1−p9q9[e2f]^tot)−κ11q8−κ12p5q5q8

(B.38) dq9da=κ10m(1−p8)p10q9q10[e2f]^tot+κ13−κ14q9−κ15p1q1q9

(B.39) dq10da=κ10m(p8−1)q10(1−p9q9[e2f]^tot)+κ16−κ17q10

(B.40) $$q_{2}=p_{c}-q_{5}$$

(B.41) $$q_{4}=\frac{p_{e_{1}}q_{2}}{q_{2}+\frac{\kappa_{5}+\kappa_{6}}{\kappa_{4}}},\;q_{3}+q_{4}=p_{e_{1}}$$

(B.42) $$q_{7}=\frac{p_{e_{2}}q_{5}}{q_{5}+(\kappa_{8}+\kappa_{9})},\;q_{6}+q_{7}=p_{e_{2}}$$

(B.43) $$\frac{{\mathrm{dp}}_{1}}{\mathrm{da}}=-\kappa_{3}(1-p_{1})-\kappa_{15}(1-p_{9})p_{1}q_{9}$$

(B.44) dp9da=−κ10m(1−p8)p10p9q10[e2f]^tot−(1−p9)(κ14+κ15p1q1)

(B.45) dp10da=−κ10m(p8−1)p10(1−p9q9[e2f]^tot)−κ17(1−p10)

(B.46) $$p_{2}=p_{5}$$

(B.47) $$p_{4}=p_{2}p_{3}$$

(B.48) $$p_{7}=p_{2}p_{6}$$

If we set $p_{i}=1$ in Eqs. (B.35), (B.36), (B.37), (B.38), (B.39), (B.40), (B.41), (B.42), (B.43), (B.44), (B.45), (B.46), (B.47), (B.48), we recover the mean-field model proposed in Bedessem and Stephanou (2014). This limit allows us to determine the parameter values of the stochastic model (see Alarcón, 2014, de la Cruz et al., 2015 for details). This equivalence is shown in Table B2. The parameter values of the mean-field model (Bedessem and Stephanou, 2014) are given in Table B3.
